# Influences of lone-pair electrons on directionality of hydrogen bonds formed by hydrophilic amino acid side chains in molecular dynamics simulation

**DOI:** 10.1038/s41598-017-16203-w

**Published:** 2017-11-20

**Authors:** Tomotaka Oroguchi, Masayoshi Nakasako

**Affiliations:** 1 Department of Physics, Faculty of Science and Technology, Keio University, 3-14-1 Hiyoshi, Kohoku-ku, Yokohama, 223-8522 Japan; 2 RIKEN SPring-8 Center, 1-1-1 Kohto, Sayo, Sayo-gun, Hyogo, 679-5148 Japan

## Abstract

The influence of lone-pair electrons on the directionality of hydrogen bonds that are formed by oxygen and nitrogen atoms in the side chains of nine hydrophilic was investigated using molecular dynamics simulations. The simulations were conducted using two types of force fields; one incorporated lone-pair electrons placed at off-atom sites and the other did not. The density distributions of the hydration water molecules around the oxygen and nitrogen atoms were calculated from the simulation trajectories, and were compared with the empirical hydration distribution functions, which were constructed from a large number of hydration water molecules found in the crystal structures of proteins. Only simulations using the force field explicitly incorporating lone-pair electrons reproduced the directionality of hydrogen bonds that is observed in the empirical distribution functions for the deprotonated oxygen and nitrogen atoms in the *sp* ^2^-hybridization. The amino acids that include such atoms are functionally important glutamate, aspartate, and histidine. Therefore, a set of force field that incorporates lone-pair electrons as off-atom charge sites would be effective for considering hydrogen bond formation by these amino acids in molecular dynamics simulation studies.

## Introduction

Hydrogen bonds (H-bonds) play an important role in the various biomolecular processes of proteins such as protein folding, molecular recognition, and enzymatic reactions^1–3^. Therefore, for simulation studies investigating the mechanisms of protein functions at an atomic level, it is essential to correctly simulate H-bond geometry. Statistical analyses of the X-ray crystal structures of proteins have revealed that the H-bond directionality, and in particular the nonlinear geometry of the H-bond at an acceptor atom, is a crucial parameter for understanding the intra- and intermolecular interactions formed by proteins^4–9^. Theoretical studies using quantum mechanical (QM) calculations have demonstrated that such H-bond directionality arises as a result of an anisotropic electron density distribution in the acceptor atom, such as lone-pair (LP) electrons^8,10,11^.

Although QM calculations can help to describe the H-bond geometry accurately, it is difficult to apply them to large molecular systems such as proteins. Therefore, the most widely used method that is currently employed to trace the molecular processes of proteins is molecular dynamics (MD) simulation^12^. Most MD simulation studies use molecular mechanical force fields based on atom-centered charges (monopole electrostatic)^13,14^. These force fields have been carefully developed, and to date, MD simulations using them have made great contributions to the studies of folding and functional dynamics of proteins^12–17^. However, because LP electrons are neglected in the usual force fields, the investigation on whether current MD simulations can reproduce experimentally observed H-bond geometries would be still necessary in order to enhance the usefulness of MD.

To date, several groups have pioneered the force fields that incorporate LP electrons either in an explicit or implicit form. In the explicit form, LP electrons are incorporated as off-atom charge sites into both fixed charge^10,18,19^ and polarizable force fields^20–22^. In the implicit form, multipole electrostatic including dipole and quadrupole terms of atomic interactions is introduced into both non-polarizable^23,24^ and polarizable force fields^25^. These studies have shown that the inclusion of LP electrons improves the agreement of MD simulations with QM calculations and experimental thermodynamic quantities. In spite of these advancements, the force fields with LP electrons have been used much less often than those without LP electrons. This lack of enthusiasm might be attributed to the fact that the importance of LP electrons has not been widely recognized, partly because reports of direct comparisons between simulation results and experimental information on atomic interactions^26^ such as H-bond directionality are rare.

The hydration structures of proteins, *i.e*., the distribution of water molecules around the hydrophilic amino acid residues on the protein surfaces^27,28^, are suitable as a reference for such direct comparison. Since the relatively small water molecules move around the protein molecules with negligible steric hindrance, their stable locations around the hydrophilic residues predominantly reflect the geometrical characteristics, such as distance and directionality, of the H-bonds formed between the water molecules and the hydrophilic groups. In order to visualize the hydration structures around hydrophilic amino acids, a set of ‘empirical hydration distribution functions’ (EHDFs) were constructed from the coordinates of the oxygen atoms of water molecules that are found on protein surfaces in a large number of X-ray crystal structures^29,30^. The EHDF provides experimental evidence for the geometrical characteristics of the H-bonds formed between the hydration water molecules and the hydrophilic residues.

In this study, MD simulations were performed in order to examine whether a fixed-charge force field that incorporates LP electrons as off-atom charge sites is necessary to reproduce the H-bond geometry observed around the *sp* ^2^- or *sp* ^3^-hybridized oxygen and nitrogen atoms in the EHDF. The hydration structures of short peptides including one or two hydrophilic residues were calculated by two types of MD simulations using different force fields. While the first type used a force field assuming that only the atom-centered charges were present (MD-noLP), the second type employed a force field explicitly incorporating LP electrons as off-atom charges (MD-LP). In case of glutamate, aspartate, and histidine amino acid residues, which play important roles in molecular recognition and catalysis, only MD-LP simulations succeeded in reproducing the H-bond directionality that was observed in the EHDF. For other amino acid side chains, the two types of MD simulations gave similar results.

## Results

Here we first describe the results of restrained electrostatic potential^31^ (RESP) calculation for constructing force field parameters used in MD calculations. The trajectories of MD-LP and MD-noLP simulations were compared with the EHDF for both protonated or deprotonated oxygen and nitrogen atoms.

### RESP and MD calculations

In the RESP calculations, the oxygen and nitrogen atoms incorporating LP charge sites were polarized with negative charges present on the LP sites and positive charges located on the atom center (Table S1). The addition of LP charge site in the RESP calculations also led to small changes in the RRMS error for the QM electrostatic potential (ESP) data (Table S3). These small changes would be due to the small increments in the degrees of freedom of the tri- and tetrapeptide systems.

The hyperbolic restraint used in the present RESP calculations (0.001 au) was stronger than that used in the standard RESP procedure (0.0005 au). The polarizations obtained by the procedure using the standard restraint strength were insufficient to reproduce the hydration structures consistent with the EHDFs. In addition, the strong restraint led to small changes in the reproducibility of the QM ESP compared to the standard restraint (Table S3). Therefore, the stronger restraint strength was employed for the non-polar heavy atoms in this study.

The MD-LP simulations were executed without any errors and required computational time comparable with that necessary for MD-noLP simulations. From the 10 ns MD simulation of the Gly-Glu-Lys-Gly (GEKG) peptide, it was confirmed that the 2 ns production run was long enough to clearly visualize the time-averaged hydration structure (Supplementary Note 1 and Figs S1–4). The results also showed that the time window of 500 ps for the calculation was sufficiently long to reduce the noisy densities in the disordered bulk solvent region (Supplementary Figs S1 and S2). These results are consistent with our previous study of the hydration structure around the protein^32^. Therefore, the last 1 ns trajectory of a 2 ns production run was used to calculate the hydration structures around the peptides.

**Figure 1:**
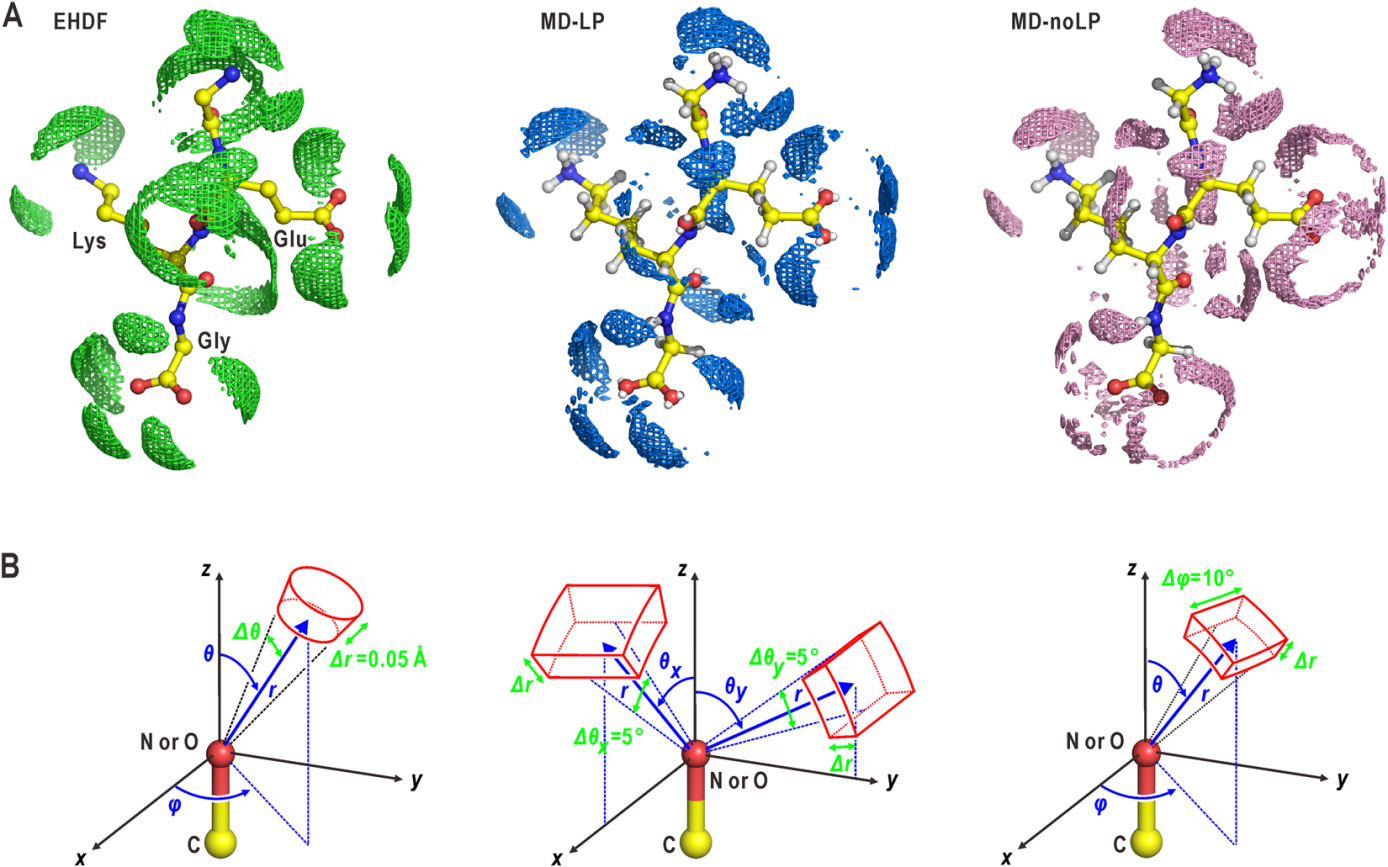
Comparison of hydration structures around the GEKG peptide. (**A**) Comparison of the solvent density maps regarding the oxygen atoms in hydration water molecules around the GEKG peptide in the EHDF (left panel) with those obtained from the MD-LP (center) and MD-noLP (right) simulations using the TIP3P model for water molecules. The density maps are contoured at 1.6 e Å^-3^. Molecular models with solvent densities are drawn using the PyMOL graphics program^60^. (**B**) Illustrations of the coordinate systems used to describe quantitatively the solvent density distribution around the oxygen or nitrogen atoms in the side chains of the amino acid residues. The volume elements drawn by using red lines were used to calculate the profiles of the distance and angular distributions of the solvent density. The parameters defining the volume elements are labeled in green. Some of these parameters were fixed to the displayed values throughout this study.

Figure 1A compares the solvent density map of EHDF around the GEKG peptide with the 1-ns averaged density distributions of oxygen atoms in hydration water molecules from the MD-LP and MD-noLP simulations. Around the oxygen atoms of the side chain in glutamate, the solvent density distributions determined from the MD-LP and EHDF were similar to each other, but differed to that calculated from the MD-noLP. In the following sections, the influences of introduced LP sites to the directionality of H-bonds are examined by quantitative analysis on solvent density distribution using the coordinate systems illustrated in Fig. 1(B).

### Hydration structure around the deprotonated *sp*^2^-hybridized oxygen atoms of glutamate side chain

**Figure 2:**
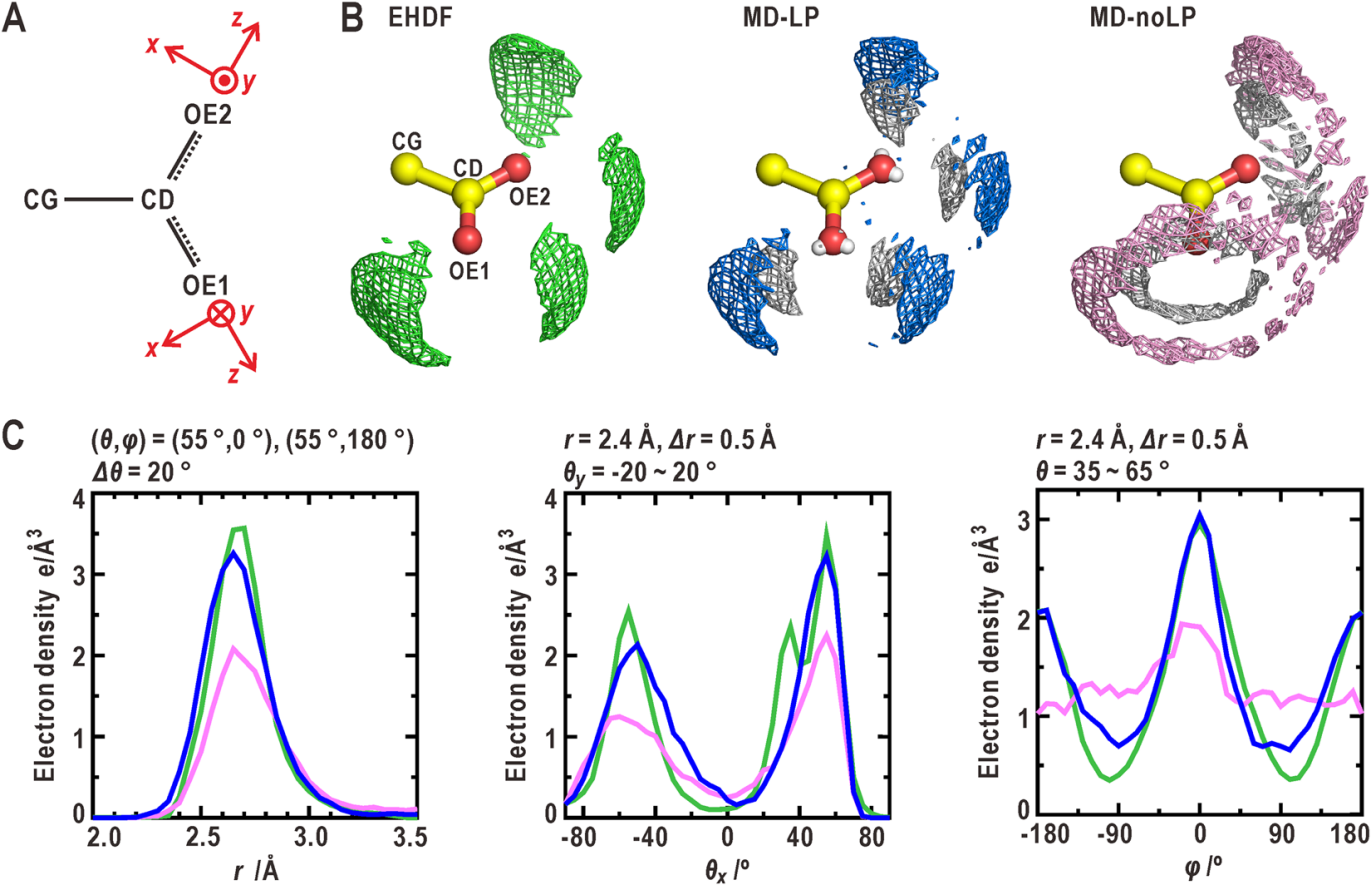
Comparison of hydration structures around the carboxyl group of glutamate. (**A**) The coordinate systems used for describing the solvent density distribution around the OE1 and OE2 atoms in the side chain. (**B**) Comparison of the solvent density maps, which display the distribution of oxygen and/or hydrogen atoms in hydration water molecules, around the OE1 and OE2 atoms among the EHDF, and MD-LP and MD-noLP simulations. The positions of the LP charge sites used in the MD-LP simulation are indicated by the small white spheres. The density maps for oxygen atoms in the EHDF (green), MD-LP (blue) and MD-noLP (pink) simulations, and for hydrogen atoms (white) are contoured at 1.6 and 0.4 e Å^-3^, respectively. These schemes are used in the subsequent illustrations of solvent density maps. (**C**) The profiles of distance (left panel), *θ*_*x*_ (center), and *φ* (right) distributions of oxygen atoms in hydration water molecules. The green, blue, and pink lines are the profiles from the EHDF, MD-LP, and MD-noLP simulations, respectively. The distance distribution is calculated by averaging the four clusters of the solvent densities of oxygen atoms. The *θ*_*x*_- and *φ-*distributions are averaged over the solvent densities around the OE1 and OE2 atoms. The parameters necessary for these calculations in the coordinate system shown in Fig. 1(B) are labeled at the top of each plot. The scheme and coloring for plotting the profiles is also used in the subsequent illustrations.

The distribution of the hydration water molecules around the side chain of glutamate was obtained from the MD simulation for the GEKG peptide by assuming a neutral pH condition (Fig. 2). In the MD-LP simulation, the solvent density map of oxygen atoms of water molecules resembled that of the EHDF. Each of the deprotonated oxygen atoms in the *sp* ^2^-hybridization (the OE1 and OE2 atoms) was hydrated by approximately two water molecules (Table 1). The peaks in the density map were located at a distance of 2.7 Å from the OE1 (OE2) atom along the direction from the OE1 (OE2) atom to the LP charge site ((*θ*_*x*_, *φ*) = (55°, 0°) and (*θ*_*x*_, *φ*) = (−55°, 0°)). The profiles of the distance and angular distributions were quantitatively similar to those of the EHDF (Fig. 2C and Table 1). In addition, the density peak of the hydrogen atoms in the hydration water molecules appeared between the OE1 (OE2) atom and the density peak of the water oxygen atoms lay along the direction of OE1 (OE2) -LP. This density peak indicates the directionality of the H-bonds formed between the OE1 (OE2) atom and the hydration water molecules.

**Table 1:** Comparison of peak positions of the distributions of oxygen atoms in hydration water molecules around polar side chains atoms in nine hydrophilic amino acids among the EHDF, MD-LP and MD-noLP simulations. The values in brackets are the HWHMs of the peak profiles. The spatial distributions and profiles are shown in Figs 2–5 and Supplementary Figs S5–S10.

Polar atoms (configuration)/amino acid (state)	Type of distribution and total amount	Peak positions		
		EHDF	MD-LP	MD-noLP
COO^−^ group (OE1 and OE2)/glutamate	distance/Å	2.70 (0.14)	2.65 (0.17)	2.65 (0.18)
	angle *θ*_*x*_/°	−55 (14), 55 (18)	−50 (21), 55 (12)	−60 (22), 55 (13)
	angle *φ*/°	−175 (31), 0 (41)	−175 (34), 0 (31)	−20 (30)
	Total amount^a^	4.0	4.4	4.3
COO^−^ group (OD1 and OD2)/aspartate	distance/Å	2.70 (0.14)	2.65 (0.14)	2.65 (0.15)
	angle *θ*_*x*_/°	−55 (13), 55 (9)	−50 (17), 45 (14)	−55 (29), 40 (19)
	angle *φ*/°	−175 (35), 0 (33)	−180 (33), 0 (30)	30 (19)
	Total amount^a^	4.0	4.4	4.1
ND1/histidine (HIE)	distance/Å	2.70 (0.14)	2.65 (0.12)	2.85 (0.18)
	angle *θ*_*x*_/°	0 (12)	0 (13)	10 (23)
	angle *θ*_*y*_/°	5 (16)	0 (19)	−80, 80
	Total amount^b^	1.0	1.3	1.7
NE2/histidine (HIE)	distance/Å	2.75 (0.14)	2.90 (0.17)	2.95 (0.19)
	angle *θ*_*x*_/°	0 (15)	5 (18)	5 (20)
	angle *θ*_*y*_/°	5 (12)	0 (18)	0 (21)
	Total amount^b^	1.0	0.7	0.6
ND1/histidine (HID)	distance/Å		2.85 (0.15)	2.90 (0.17)
	angle *θ*_*x*_/°		0 (16)	10 (20)
	angle *θ*_*y*_/°		0 (17)	−5 (20)
	Total amount^b^		0.9	0.5
NE2/histidine (HID)	distance/Å		2.65 (0.11)	2.80 (0.17)
	angle *θ*_*x*_/°		0 (14)	−5 (36)
	angle *θ*_*y*_/°		0 (21)	−80, 80
	Total amount^b^		1.4	1.6
OH (*sp* ^2^)/tyrosine	angle *θ*_*x*_/°	−60 (7), 60 (6)	−60 (13), 60 (10)	
	distance (OH-HH)/Å	2.65 (0.13)	2.75 (0.16)	
	angle *θ*_*y*_ (OH-HH)/°	−5 (22)	−5 (16)	
	Total amount on the OH-HH side^c^	1.0	0.9	
	distance (OH-LP)/Å	2.65 (0.13)	2.80 (0.17)	
	angle *θ*_*y*_ (OH-LP)/°	−5 (22)	10 (42)	
	Total amount on the OH-LP side^c^	1.0	0.6	
OH (*sp* ^3^)/tyrosine^g^	angle *θ*_*x*_/°		−60 (15), 65 (8)	−65 (13), 65 (78)
	distance/Å		2.75 (0.16)	2.75 (0.15)
	angle *θ*_*y*_/°		0 (18)	0 (14)
	Total amount on the OH-HH side^c^		0.9	0.9
OG/serine	distance/Å	2.70 (0.14)	2.75 (0.16)	2.70 (0.14)
	angle *θ*_*x*_/°	−45 (13), 75 (11)	−55 (10), 75 (13)	−55 (11), 70 (12)
	angle *φ* (α)/°		−140 (38), 10 (22)	−140 (40), 10 (20)
	Total amount (α)^d^		1.9	2.0
	angle *φ* (β)/°		−90 (20), 100 (70)	−90 (20), 100 (75)
	Total amount (β)^d^		1.8	1.8
	angle *φ* (γ)/°		−130 (27), −60 (31), 90 (22)	−120 (21), 90 (46)
	Total amount (γ)^d^		1.8	1.8
	angle *φ* (average)/°	−90 (21), 10 (41), 90 (18)	−100 (36), 10 (15), 90 (23)	−100 (26), 10 (15), 100 (19)
	Total amount (average)^d^	3.0		
OG1/threonine	distance/Å	2.70 (0.11)	2.75 (0.14)	2.70 (0.13)
	angle *θ*_*x*_/°	−50 (10), 65 (8)	−50 (9), 65 (9)	−50 (11), 70 (9)
	angle *φ* (α)/°		−145 (34), −10 (17)	−140 (26), 0 (14)
	Total amount ^d^		2.0	2.0
	angle *φ* (β)/°		−100 (17), 110 (42)	−100 (17), 105 (44)
	Total amount^d^		1.8	1.9
	angle *φ* (γ)/°		−120 (27), −35 (29), 110 (17)	−120 (31), −35 (29), 110 (18)
	Total amount^d^		2.0	2.0
	angle *φ*(average)/°	−100 (15), −5 (31), 110 (25)	−110 (26), −10 (17), 110 (19)	−110 (25), −10 (17), 110 (22)
	Total amount^d^	3.0		
NZ (NH_3_^+^)/lysine	distance/Å	2.80 (0.18)	2.85 (0.13)	2.85 (0.13)
	angle *θ*/°	75 (12)	70 (15)	70 (15)
	angle *φ*/°	−120 (21), 0 (20), 120 (21)	−120 (16), 0 (22), 120 (19)	−120 (16), 0 (20), 120 (20)
	Total amount^e^	3.0	3.3	3.3
NE, NH1, NH2/arginine	distance (NE)/Å	2.85 (0.18)	2.85 (0.15)	2.90 (0.14)
	angle *θ*_*x*_ (NE)/°	0 (17)	0 (25)	0 (24)
	distance (NH1)/Å	2.95 (0.20)	2.85 (0.16)	2.85 (0.16)
	angle *θ*_*x*_ (NH1)/°	−50 (6), 60 (22)	−50 (7), 65 (26)	−50 (7), 65 (27)
	distance (NH2)/Å	2.90 (0.21)	2.85 (0.14)	2.85 (0.15)
	angle *θ*_*x*_ (NH2)/°	−65 (35), 60 (25)	−65 (28), 65 (73)	−65 (32), 65 (24)
	Total amount^f^	5.0	4.4	4.5
ND1/tryptophan	distance/Å	2.90 (0.15)	2.90 (0.16)	2.90 (0.15)
	angle *θ*_*x*_/°	0 (13)	0 (17)	0 (16)
	angle *θ*_*y*_/°	5 (12)	0 (18)	0 (18)
	Total amount^g^	1.0	0.8	0.8

In order to calculate the total density of hydration water molecules around the polar groups, the volumes were defined by the following parameters;

^a^(*r* = 2.2‒3.2 Å, *θ*_*x*_ = 30‒70°, *φ* = −180‒180°),

^b^(*r* = 2.2‒3.2 Å, *θ*_*x*_ = −25‒25°, *θ*_*y*_ = −180‒180°),

^c^(*r* = 2.2‒3.2 Å, *θ*_*x*_ = −20‒20°, *θ*_*y*_ = −40‒40°),

^d^(*r* = 2.2‒3.2 Å, *θ*_*x*_ = 45‒80°, *φ* = −180‒180°),

^e^(*r* = 2.2‒3.4 Å, *θ*_*x*_ = 40‒90°, *φ* = −180‒180°),

^f^(total hydration density of the five clusters in the range of *r* = 2.2‒3.4 Å, *y* = −2‒2 Å),

^g^(*r* = 2.4‒3.4 Å, *θ*_*x*_ = −40‒40 °, *θ*_*y*_ = −40‒40 °).

In the MD-noLP simulation, the solvent density map around the OE1 (OE2) atom was different from those of the EHDF and the MD-LP simulation. The density maps of both the oxygen and hydrogen atoms in the hydration water molecules were distributed in toroidal shapes, the centers of which were located in the CD-OE1 (CD-OE2) direction. The density in the *φ*-distribution was almost uniform, except for a small maximum at approximately *φ* = 0°. However, the total number of hydration water molecules was two, similar to the MD-LP simulation. In addition to the case of glutamate, around the side chain of aspartate, the MD-LP simulation gave a solvent density map similar to that obtained from the EHDF, while the MD-noLP simulations did not (Supplementary Note 2 and Fig. S5, and Table 1).

### Hydration structures around the deprotonated nitrogen atoms in the *sp*^2^-hybridization of the histidine side chain

The imidazole ring of histidine has two nitrogen atoms, ND1 and NE2, in the *sp* ^2^-hybridization. Since each of the nitrogen atoms can accept a proton to form a conjugate acid, three protonation states are possible, *i.e*., di-protonated, ND1 protonated (designated as HID state) and NE2 protonated (HIE state). The population of these three states depends on the pH and/or the environment around the histidine residues in proteins. Therefore, the EHDF of a histidine side chain is the ensemble average of the three states.

**Figure 3:**
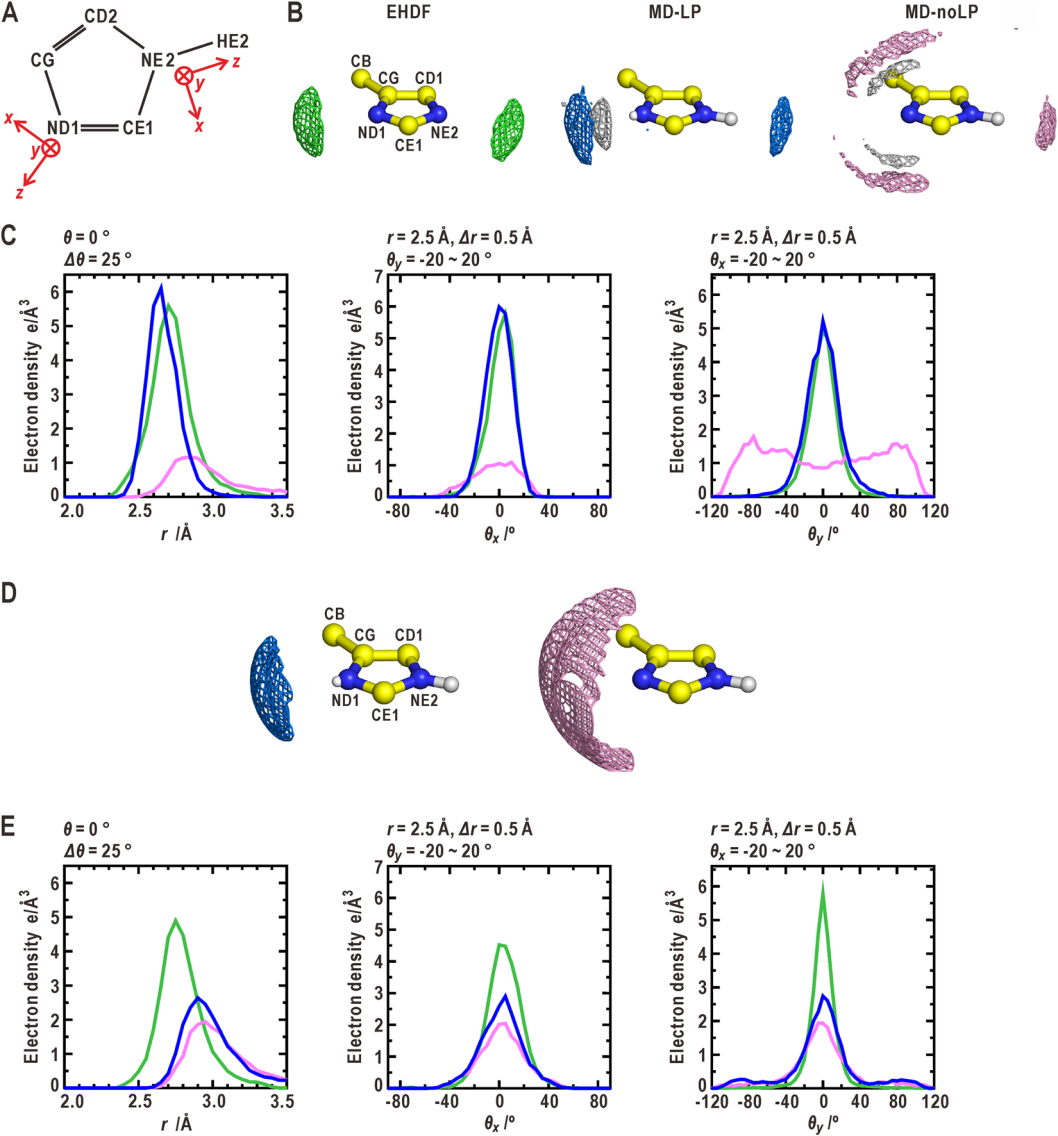
Comparison of hydration structures around the imidazole ring of histidine. (**A**) The coordinate systems used for the descriptions of the solvent density distributions around the ND1 and NE2 atoms of the imidazole ring. (**B**) Comparison of the solvent density maps around the imidazole ring in the HID state among the EHDF, MD-LP and MD-noLP simulations. (**C**) The distance (left panel), *θ*_*x*_ (center), and *θ*_*y*_ (right) distributions of oxygen atoms in hydration water molecules around the ND1 atom. (**D**) Potential maps around the ND1 atom in the HIE state calculated from the force fields with (left) and without (right) incorporating LP electrons. The potential map in the left panel is contoured at +2.0 kcal·mol^−1^ from the minimum (−13.4 kcal·mol^−1^), while the map in the right pane is contoured at +1.0 kcal·mol^−1^ from the minimum (−7.7 kcal·mol^−1^). (**E**) The distance (left panel), *θ*_*x*_ (center), and *θ*_*y*_ (right) distributions of oxygen atoms in hydration water molecules around the NE2 atom.

The MD simulations were applied separately for the HIE (Fig. 3) and HID states (Supplementary Note 3 and Fig. S6). In the solvent density map obtained from the MD-LP simulation for the HIE state, a single density peak of oxygen atoms in hydration water molecules appeared along the direction from the deprotonated ND1 atom to the LP charge site (*θ*_*x*_ = *θ*_*y*_ = 0°) as observed in the EHDF. The profiles of the distance and angular distributions of the solvent density map were consistent with those of the EHDF. Therefore, as well as the EHDF, the ND1 atom was hydrated by approximately one water molecule (Table 1). In addition, a single density peak of hydrogen atoms in the hydration water molecules was localized between the ND1 (NE1) atom and the peak of the water oxygen atom, indicating the directionality of the H-bond formed between the ND1 atom and the hydration water molecules. The MD-LP simulation for the HID state also produced a solvent density map resembling the EHDF (Supplementary Fig. S6 and Table 1). In contrast, for the deprotonated ND1 (NE2) atom in the HIE (HID) state, the MD-noLP simulations gave solvent density maps that differed from those of the MD-LP and EHDF. Hydration water molecules were distributed in an arc shape surrounding the nitrogen atom with two small maxima at approximately *θ*_*y*_ = ±80°.

In order to understand the causes for the differences in the solvent density distribution between the MD-LP and MD-noLP simulations, the interaction potentials between the deprotonated nitrogen atoms and the hydration water molecules were calculated. A single point charge on the center of the ND1 atom produced a wide potential valley accompanying two shallow basins in the direction of *θ*_*y*_ = ±80°. The potential valley is only able to weakly restrain the movement of hydration water molecules. In contrast, a dipole formed by the LP charge and the nitrogen atom induces a narrow potential valley that is approximately twice as much deeper than that of the point charge. This deep potential valley can trap a hydration water molecule and control the orientation of the molecule *via* dipole-dipole interaction. However, the RRMS error could not distinguish between these differences in the interaction potential, because the electrostatic contribution of the charges on the ND1 atom to the total sum of ESP data was relatively small compared to that of the charges on the whole peptide structure.

For a protonated ND1 (NE2) atom, the solvent density distributions from the MD-LP and MD-noLP simulations were similar with regard to both the peak positions and profiles in the angular distributions (Fig. 3, Supplementary Fig. S6, and Table 1). The single density peak of the oxygen atoms in water molecules appeared along the ND1-H directions (*θ*_*x*_ = *θ*_*y*_ = 0°). However, the distance distribution was inconsistent with that of the EHDF. The density was significantly lower than that of the EHDF, and therefore the protonated ND1 (NE2) atom was less hydrated in both simulations (Table 1). In addition, the peak position of the simulations at 2.90 Å was different from that of the EHDF at 2.75 Å. These findings indicate that the configuration parameters for the ND1-H (NE2-H) group are correct, but the charge values and the Lennard-Jones parameters for this group need to be improved in order to attract the hydration water molecules.

### Hydration structures around the protonated oxygen atoms in the *sp*^3^-hybridization

**Figure 4:**
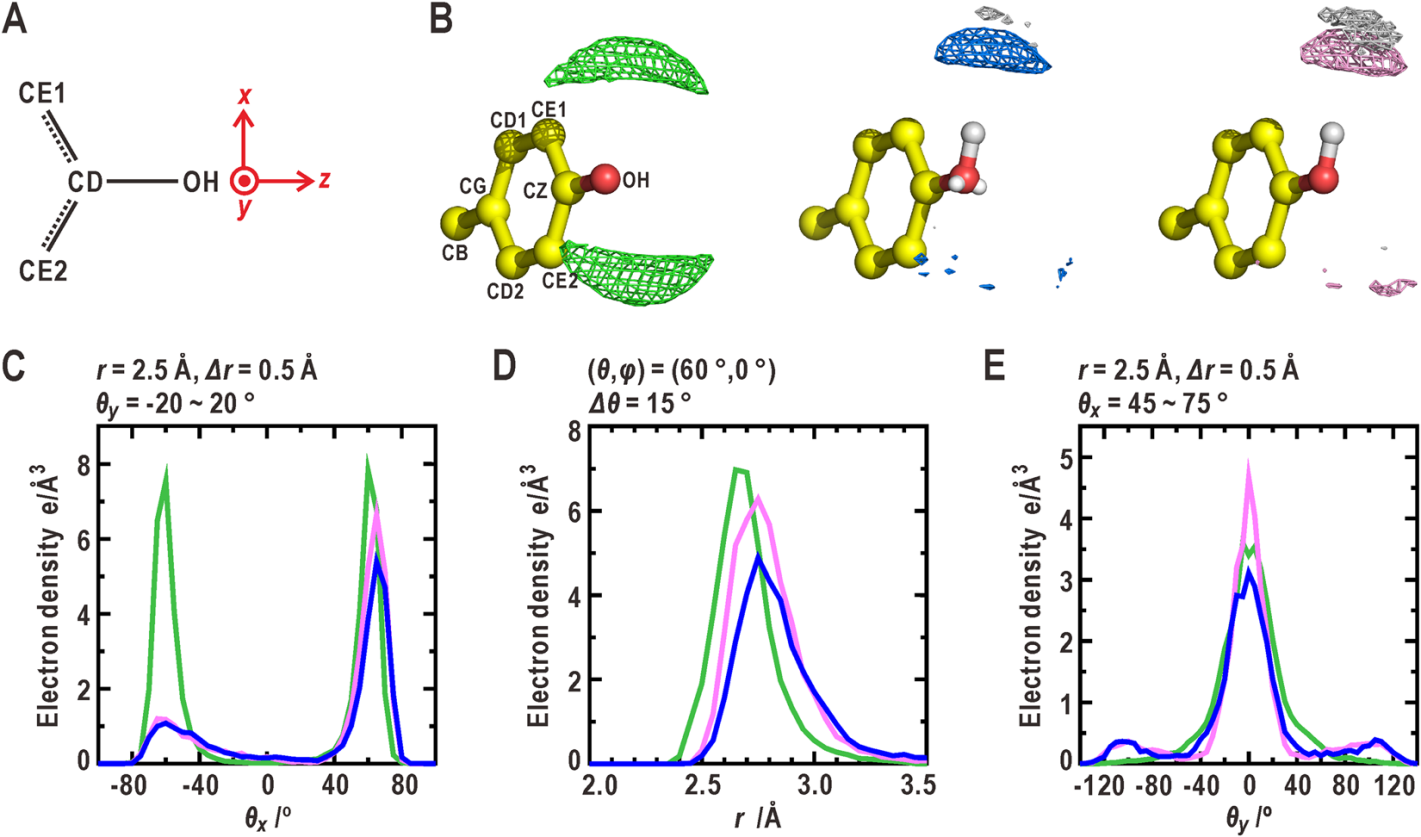
Comparison of hydration structures around the hydroxyl group of tyrosine. (**A**) The coordinate system used for the descriptions of the solvent density distributions around the O-H group in the side chain. (**B**) Comparison of the solvent density maps around the O-H group among the EHDF, MD-LP and MD-noLP simulations. The *θ*_*x*_- (**C**), distance (**D**) and *θ*_*y*_-distributions (**E**) of the oxygen atoms of hydration water molecules around the OH atom.

Between the MD-LP and MD-noLP simulations, small differences were found in the hydration structure around the *sp* ^3^-hybridized oxygen atoms in the side chains of tyrosine (Fig. 4 and Table 1) and serine (Supplementary Note 4 and Fig. S7) and threonine (Supplementary Note 4 and Fig. S8). The solvent density around the OH-HH group of tyrosine was calculated for the conformation with the dihedral angle of CE1-CZ-OH-HH fixed to 0°. A single density peak of water oxygen atoms appeared in the direction of the OH-HH bond (*θ*_*x*_ = 60°). A low solvent density appeared on the opposite side of the OH-HH bond (*θ*_*x*_ = −60°) regardless of the LP charge sites. The negative charges applied to the sites were insufficient to attract the hydration water molecules. The EHDF around the OH atom, which has two density peaks, was explained as being the ensemble average of two rotamer states of the CZ-OH bond.

The OH atom of tyrosine has been found in the *sp* ^2^-hybridization in previous reports^4,6^. Therefore, the solvent densities were also calculated using the other type of MD simulation assuming that a single LP was on the opposite side of the OH-HH bond and the other LP was involved in the π-interaction with the electrons of the aromatic ring (Supplementary Note 5 and Fig. S9). In this simulation, the single density peak of oxygen atoms in water molecules appeared on the OH-LP side, but the density of the hydration water molecules forming the peak was smaller than that on the OH-HH side.

Regarding the the *sp* ^3^-hybridized oxygen atoms in serine and threonine, the solvent density maps were calculated for three possible configurations of the side chains. These maps were almost consistent between the MD-LP and MD-noLP simulations. The map summed over the three configurations was similar to that of the EHDF with regard to its shape and the profiles of the distance and angular distributions (Supplementary Note 4, Figs S7 and S8-, and Table 1).

### Hydration structures around the protonated nitrogen atoms in the *sp*^3^- or *sp*^2^-hybridization

**Figure 5:**
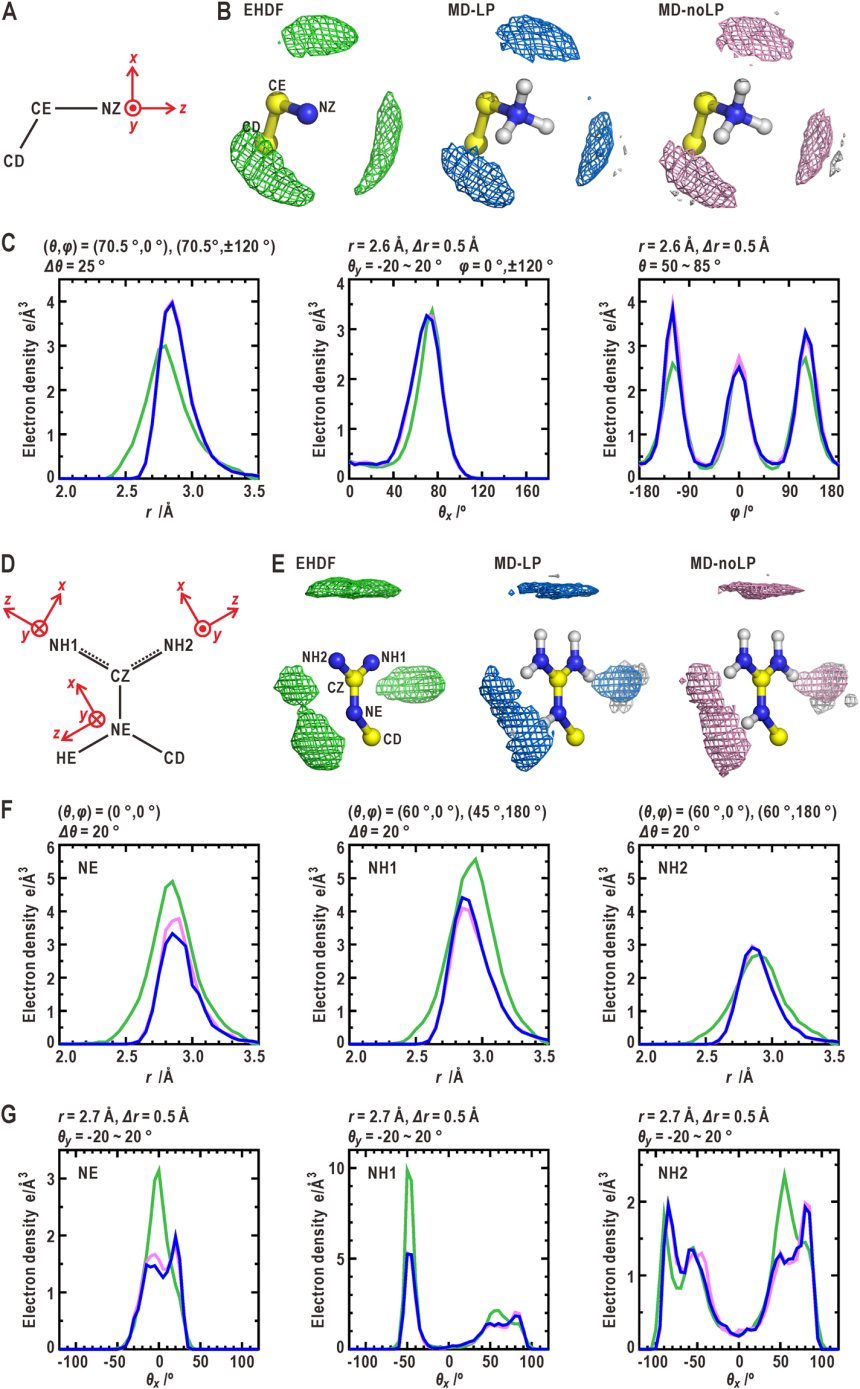
Comparison of hydration structures around the side chains of lysine and arginine. (**A**) The coordinate system used for the descriptions of the distributions in the NH_3_^+^ group of the side chain of lysine. (**B**) Comparison of the solvent density maps around the NH_3_^+^ group among the EHDF (left panel), and MD-LP (center) and MD-noLP (right) simulations. (**C**) The distance (left panel), *θ* (center), and *φ* (right) distributions of water oxygen densities around the NH_3_^+^ group. (**D**) The coordinate systems used for the descriptions of the distributions in the NH and NH_2_ groups in the side chain of arginine. (**E**) Comparison of the solvent density maps around the side chain of arginine among the EHDF, and MD-LP and MD-noLP simulations. The distance (**F**) distributions and *θ*_x_-distributions (**G**) of oxygen atoms in hydration water molecules around the protonated NE, NH1, and NH2 atoms.

Lysine has one protonated *sp* ^3^-hybridized nitrogen atom (NZ) at the tip of the side chain. Around the protonated NZ atom, both the MD-LP and MD-noLP simulations gave three density peaks of water oxygen atoms in the directions of the three NZ-H bonds at (*θ*_*x*_, *φ*) = (70°, 0°) and (70°, ± 120°), as observed in the EHDF (Fig. 5A–C and Table 1). The angular distributions had profiles consistent with those of the EHDF. On the other hand, the distance distribution was slightly different from that of the EHDF with regard to the profiles and the peak positions.

The side chain of arginine has three protonated *sp* ^2^-hybridized nitrogen atoms (NZ, NH1, and NH2). In both the MD-LP and MD-noLP simulations, the single density peak of the water oxygen atoms appeared along the directions of the N-H bond in each NH group as observed in the EHDF (Fig. 5D–G and Table 1). While the distance distribution around the protonated NH2 atom of arginine had a profile that closely resembled that of the EHDF, those of the protonated NE and NH1 atoms were slightly different with regard to the peak positions and profiles. In the *θ*_x_ distributions around the protonated NE, NH1, and NH2 atoms, the peak positions were near those of the EHDF. It is also noteworthy that the position of one density peak around NH1 was shifted by −10° from the NH1-HH11 bond axis (*θ*_*x*_ = −50°) as observed in the EHDF, and this shift was probably due to the excluded-volume effect of the CD atom near the NH1 atom in the simulations. However, the densities of hydration in the simulations were approximately the half of those in the EHDF.

Around the protonated NE1 atom in the side chain of tryptophan, the solvent density distributions from the MD-LP and MD-noLP simulations were consistent with those of the EHDF in terms of the peak positions in the distance and angular distributions (Supplementary Note 6 and Fig. S10, and Table 1). However, the densities in these distributions were approximately half of those in the EHDF.

### Influences of LP electrons on hydration structures around hydrophilic residues of proteins

To understand the influences of LPs on the hydration structures around glutamate, aspartate and histidine residues exposed on protein surfaces, MD-LP and MD-noLP simulations were applied to Sec7 domain of guanine-releasing factor^33^ (Sec7), ribonuclease Sa^34^ (RNase-Sa) and cytoplasmic portion of histidine-kinase protein^35^ (HK) as examples.

**Figure 6:**
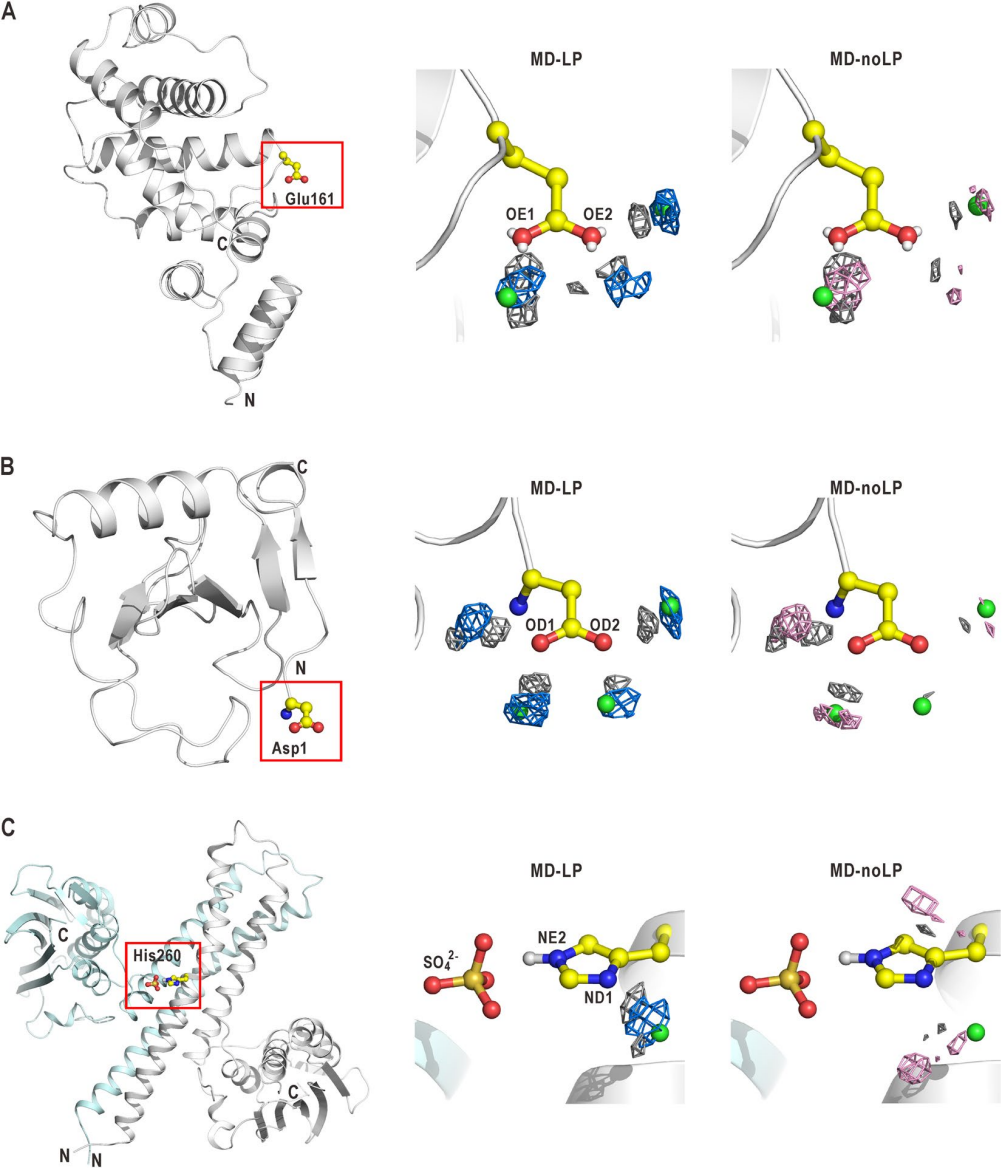
Hydration structures around glutamate and histidine residues in proteins. Hydration structures around Glu161 of sec7 (**A**), Asp1 of RNase-Sa (**B**), and His260 of HK (**C**) in the crystal structure, MD-LP and MD-noLP simulations. Left panels illustrate the locations of the residues (ball-and-stick models) in the whole structures (ribbon models). Center and right panels display magnified views of the residues with crystal water sites (green spheres), the density maps of oxygen atoms (blue mesh for MD-LP and pink for MD-noLP) and maps of hydrogen atoms (grey mesh) in hydration water molecules. In the case of HK, a sulfate ion interacting with His260 is displayed.

In the crystal structure of Sec7, a crystal water molecule hydrates the OE2 atom of the functionally important residue Glu161 along the direction from this atom to the LP charge site (Fig. 6A). Only the MD-LP simulation gave a density peak of water oxygen atoms near the crystal water site. For the OD2 atom of the residue Asp1 of RNase-Sa, the solvent density peaks appeared at the locations of the two crystal water molecules hydrating the OD2 atom only in the MD-LP simulation (Fig. 6B).

In HK, one crystal water molecule is located near the ND1 atom of His260 acting as a phosphorylation site (Fig. 6C). The NE2 atom would be protonated because of the interaction with a sulfate ion near this atom, while the ND1 atom would be deprotonated considering the crystallization condition of pH 6.5 and pKa of histidine. A crystal water molecule formed one H-bond with the ND1 atom. In the MD-LP simulation, the single density peak was observed at the location of this crystal water site, but the MD-noLP simulation gave two density peaks at the locations different from that of the crystal water site. These results indicate that the MD-LP simulation was suitable to reproduce the locations of crystal water sites around the polar atoms in the side chains of glutamate, aspartate and histidine residue on the surface of proteins.

## Discussion

MD simulations with the force field explicitly incorporating LP electrons gave solvent density distributions that were similar to those experimentally obtained around the deprotonated *sp* ^2^-hybridized oxygen and nitrogen atoms in the side chains of glutamate, aspartate, and histidine in peptides (Figs 2, 3, and Supplementary Figs S5 and S6) and proteins (Fig. 6). On the other hand, the incorporation of LP charge sites in the force field had small influence on the hydration structures around the protonated *sp* ^3^-hybridized oxygen atoms present in the side chains of tyrosine, serine, and threonine (Fig. 4, Supplementary Figs S7 and S8). Here we discuss the influence of LP electrons on the hydration structures around the side chains of amino acids, the usefulness of EHDFs in the validation of force field parameters, and the future improvements of the charge set determined in this study are discussed in this section.

We first discuss the influence of LP electrons on the hydration structures around the side chains of amino acids. QM studies on the energetics of the H-bond formation of the type X-H···O=C (where X represents a nitrogen or oxygen atom) have revealed that the most stable H-bond is formed along the direction from the center of the acceptor atom to the chemically intuitive positions of the LP electrons.^8,10^ Such H-bond directionality found in the QM studies of *sp* ^2^-hybridized acceptor atoms is consistent with the directionalities observed in the EHDF and the MD-LP simulations (Figs 2, 3, Supplementary Figs S5 and S6). Therefore, the simple force field models, in which the LP electrons are approximated as single point charges at off-atom sites, are deem to be sufficient to reproduce the directionality of H-bonds expected from the QM calculations. In terms of classical electrostatics, a pair of an LP charge site and an acceptor atom forms an electric dipole along the acceptor-LP charge site direction. Subsequently, H-bonds are preferentially formed when the O-H bond of the hydration water molecule is in line with the acceptor-LP charge site direction.

Between the MD-LP and MD-noLP simulations, there are a few differences in the hydration structures around the O-H group, in which the oxygen atom is in the *sp* ^3^-hybridization and has two LP charge sites (Fig. 4, Supplementary Figs S7 and S8). In the cases, the hydration water molecules were arranged according to the electrostatic interactions with the dipole formed by the O-H pair. On the other hand, prominent solvent densities were missing around the LP sides of each O-H group. This can be partly explained by the small polarization of the OH atom that was induced by the charges given to this atom (−0.02 e) and the two LPs (−0.19 e). The present results determined for the *sp* ^2^-hybridized glutamate, aspartate, and tyrosine suggest that positive charges larger than + 0.15 e for polar atoms and negative charges smaller than −0.30 e for the LP charge site are necessary to reproduce the hydration structure consistent with the EHDF (Table S1). This result is also consistent with the QM study^10,11^ which revealed a small energy dependence on the geometry of the H-bond formed between a *sp* ^3^-hybridized oxygen atom of methanol and the O-H group of a hydration water molecule.

In comparison with the previous fixed-charge force field including LP charge sites, the charges on polar atoms incorporating LP sites in the present study (Table S1) were more polarized due to the restraint stronger than that used in the standard RESP procedure. As mentioned in the results, the polarizations obtained by the procedure using the standard restraint strength were insufficient to reproduce the hydration structures consistent with the EHDFs (Figs 3E and 5C,F).

The directionality of H-bonds found in the EHDF is also observed experimentally in protein-protein^9,36^ and protein-ligand interactions^37,38^, as well as those in the secondary^4,7,26^ and tertiary protein structures^6,8^. Therefore, a correct description of the H-bond geometry would enhance the usefulness of MD simulations for the studies of the various molecular processes of proteins such as folding and molecular recognition. On protein surfaces, MD-LP simulations can reproduce the locations of crystal water sites around deprotonated nitrogen and oxygen atoms in the *sp* ^2^-hybridization better than MD-noLP simulations (Fig. 6).

The incorporation of the LP charge sites of oxygen and nitrogen atoms in the force field only causes a small increase in the degrees of freedom in MD simulations of proteins. Because the degrees of freedom is primarily dominated by a large number of water molecules, the computational cost necessary to execute an MD-LP simulation is almost the same as that for an MD-noLP simulation. Thus, a fixed-charge force field including LP charge sites would be one of items for the tracing the various molecular processes of proteins by MD simulations.

Next, we discuss usefulness of the EHDFs in validating force field parameters. The EHDFs provide the experimental information on the atomic interactions between proteins and water molecules. Therefore, the direct comparisons between the EHDFs and the hydration structures calculated from the MD simulations enabled us to examine two types of force field with respect to the H-bond geometry as demonstrated in this study (Figs 1–4 and Supplementary Figs S5–S10). In addition, the EHDF implicitly contains the information about the positions of hydrogen atoms of proteins. For example, the simulations for lysine were able to reproduce the density peaks of solvent densities in the directions of each N-H bond as observed in the EHDF. The locations of the density peaks in the EHDF reflect the configurations of the NH_3_^+^ group, and then the consistency regarding the locations between the EHDF and the MD simulations implies that the EHDF is a useful reference for validating the configuration parameters of hydrogen atoms in the force field. In this regard, the EHDF also acts as a reference to validate the chemically intuitive positions of the LP charge sites that are assumed in the force field.

Finally, we discuss the future improvements of the charge set determined in this study. In this study, we focused on the determination of charges on the side chains of the hydrophilic amino acid residues. However the improvement of the charges on the main chains should be also necessary for the development of useful force field in a future study, because several theoretical studies have postulated that the anisotropic electron density distributions in the atoms of peptide bonds play certain roles in conformational changes of peptides and proteins^26,39–41^. Although the hydration structure around main chain depends on the conformations^29,42,43^ and environmental structures, EHDFs of main chains exposed to solvent would be constructed from crystal water sites around peptide bonds exposed well to solvent.

The distance distributions around some protonated nitrogen atoms show significant differences from those of EHDFs in terms of the peak positions and the density of the hydration water molecules (Figs 3,5, Supplementary Fig. S6, and Table 1). A probable cause for this difference is the insufficient polarization of protonated nitrogen atoms, that would be partly caused by the RESP procedure used for the charge determination in this study. In the procedure, the ESP was calculated at the 6–31 G* level for the implicit treatment of atomic polarizations induced by the hydration water molecules^44^. For a more accurate estimation of water-induced polarization, the QM calculations with explicit water molecules would be necessary in addition to a slight modification of the Lennard-Jones parameters for protonated nitrogen atoms in the future.

In recent years, the number of protein crystal structures solved at ultra-high resolution beyond 0.8 Å has gradually increased^45^. These crystal structures visualize both the anisotropic distributions of electron densities of protein atoms and provide the positions of hydrogen atoms. The anisotropic distribution of electron densities would provide hints to estimate the positions of LP charge sites and the charges in the polar groups with hydrogen atoms, and be also useful for examining the effectiveness of a polarizable force field with multipole electrostatics, which can treat atomic interactions more accurately. In addition, the positions and electron densities of hydrogen atoms allow us to construct a more accurate EHDF.

In conclusion, this study demonstrates that simple models incorporating the LP charge sites are effective to reproduce the directionality of H-bonds formed by the deprotonated *sp* ^2^-hybridized oxygen and nitrogen atoms in the side chains of glutamate, aspartate, and histidine. Because these side chains play important roles in protein functions such as molecular recognition and catalysis, the force field with LP charge sites would be one of important items for MD simulation studies of the various molecular processes in proteins.

## Methods

### Brief description of EHDF

EHDFs around the side chains of glutamate, aspartate, lysine, arginine, histidine, tyrosine, serine, threonine, and tryptophan (see Supplementary Note 7) were constructed from 401,794, 423,622, 151,717, 228,496, 69,436, 128,362, 174,132, 191,684, and 18,086 crystal water sites, respectively, in 18,007 crystal structures that were refined using the diffraction data collected below 150 K and at resolutions better than 2.2 Å^29^. The EHDFs were constructed from hydration water molecules bound to polar atoms of hydrophilic residues, most of which are exposed to solvent channels in crystals. Because the solvent channels retain the fluidity enough to ensure the penetration of small molecules, water molecules would be able to freely move as well as those in aqueous solutions. Therefore, the EHDFs provide the solvent density distributions that would be almost equivalent to the ensemble-averaged hydration structures around oxygen and nitrogen atoms of these amino acids in aqueous solutions. Most of the major peaks in EHDFs appear in the N-H, O-H, N-LP, or O-LP directions. The EHDF of each atom is normalized so that the sum is equal to the total number of electrons of oxygen atoms in the water molecules that can possibly form H-bonds with that atom (Table S2).

Previous studies^29,42,43^ have shown that the locations of the oxygen atoms of water molecules that hydrate the peptide bond strongly depend on the conformations of the main chain. Therefore the EHDF around a peptide bond is averaged over the various hydration structures that exist around the different main-chain conformations^29^. This particular characteristic of the EHDF around the peptide bond is expected to make comparisons with MD simulations complicated. Therefore, the main focus of this study is to investigate the hydration structures around the side chains of amino acids. Furthermore, the LPs on the sulfur atoms of cysteine and methionine have not been considered in the simulations because their EHDFs have not been constructed.

### Construction of force field and MD simulation

The MD simulations for calculating the hydration structures of nine hydrophilic amino acids were carried out in four steps. In the first step, short peptides with the sequences Gly-Glu-Lys-Gly (GEKG), Gly-Asp-Lys-Gly (GDKG), Gly-Glu-Arg-Gly (GERG), Gly-His-Gly (GHG), Gly-Tyr-Gly (GYG), Gly-Ser-Gly (GSG), Gly-Thr-Gly (GTG), and Gly-Trp-Gly (GWG) were constructed. For charged residues, a pair of single acidic and basic residues was tandemly arranged for the neutralization of a MD system without ions. The main chains of the peptides in β-strand conformations were taken from crystal structures (the accession codes in the Protein Data Bank: 1WZ1^46^ and 3EIS^47^) to expose the side chains to solvent with large accessible solvent area. Since the objective of this study was to investigate the hydration structures around the side chains, the rotamer states of the side chains were selected so as to maximize the solvent accessible surface of the targeted side chains.

The LP electrons were implemented in the AMBER force field^13^, ff99SBildn^48^, as off-atom charge sites added to atom-centered charges. The positions used were those of the LP charge sites determined in an earlier study^10^. Both the O-LP and N-LP distances were 0.35 Å. For the *sp* ^2^-hybridized oxygen atom, two LP charge sites were placed in the plane of the hybridization orbital and subsequently, both the C-O-LP and LP-O-LP angles were set to 120.0°. For the *sp* ^2^-hybridized nitrogen atom, a single LP charge site was placed in the plane of the orbital and the C-N-LP angle was set to 121.5°. Two LP charge sites were placed in the tetrahedral configuration on the protonated *sp* ^3^-hybridized oxygen atoms in the side chains of tyrosine, serine, and threonine. In case of the tyrosine side chain with *sp* ^2^ hybridization, the HH-OH-CZ and HH-OH-LP angles were set to 120.0°. The mass and Lennard-Jones well depth for the LP sites were set to 0. The positions of the LP sites relative to that of any polar group were fixed in simulations^49^.

In the second step, the charges of the atoms and the LP sites of the constructed tri- and tetrapeptides were determined by the RESP method^31^. The ESP around the peptides were calculated using the Hartree-Fock scheme with the standard 6–31 G* basis set and tight self-consistent field convergence criteria using the program GAUSSIAN03^50^. The ESP points lay on the surface outside the van der Waals radii of the atoms. The values for the number of surface and the surface density of ESP points were set to 4 and 1, respectively.

In the RESP calculations for the models without the LP charge sites, the charges of the heavy atoms (except for oxygen and nitrogen) were hyperbolically restrained to be close to zero with the restraint strength of 0.001 au. In the RESP calculations for the models with LP charge sites, the charges of heavy atoms were restrained with the same strength, with the exception of the oxygen atoms of COO^−^ groups and nitrogen atoms. The restraint used here was stronger than that used in the standard RESP procedure for amino acids^44,51^ (the standard restraint strength = 0.0005 au). The charges of chemically equivalent atoms such as the hydrogen atoms of a methyl group were constrained to have same values. The determined charges for the side chains of nine amino acids are provided in Table S1. The qualities of the RESP calculations were measured by the relative root-mean-square (RRMS) errors for the ESP data (Table S3). The charges of glutamate and lysine residues were also determined by using the standard RESP protocol (Table S4). In this protocol, the multi-conformational RESP fitting was applied to the dipeptide in which each target amino acid was blocked by the acetyl and *N*-methyl groups. Based on comparison of these two protocols regarding the charge values and hydration structures calculated from the MD simulations of the GEKG peptide calculated, we decided to employ the present RESP protocol for the determination of charges in the tri- or tetrapeptides (Supplementary Note 8 and Figs S11–S13).

In the third step, the two types of force fields determined in the previous step were utilized to sample the positions of the hydration water molecules around each peptide model by MD simulations. Each peptide was first immersed in a water box with the dimensions of 59 × 59 × 59 Å^3^ that was composed of ca. 5550–5650 water molecules of the TIP3P model^52^. The TIP4P/Ew^53^, TIP5P^18^, and SPC/E^54^ water models were compared with the TIP3P model regarding the reproducibility of the EHDFs in both MD-LP and MD-noLP simulations as described in Supplementary Note 9 and illustrated in Supplementary Figs S14 and S15 with Table S5. From these comparisons, we selected the TIP3P model over the other models. Under the periodic boundary condition, the dimensions of the water box were wide enough to separate the peptide from its periodic images by more than 30 Å. The MD simulations were conducted at 293 K using the AMBER software^55^. The electrostatic interactions were treated using the particle mesh Ewald method^56^, with the real space cutoff of 10 Å. The Lennard-Jones interactions were truncated beyond 10 Å with continuum model corrections according to the protocol used in AMBER. Bonds involving hydrogens were constrained by the SHAKE method^57^, and the time step was set to 0.002 ps. For controlling the temperature, the Andersen temperature coupling scheme^58^ was used.

Each MD system was first subjected to an energy minimization of 10,000 steps. The temperature of the system was then raised from 10 to 293 K by an NPT run of 300 ps under 1 atm. After equilibration, the dimensions of the system were 56 × 56 × 56 Å^3^. Finally, we conducted a several-ns production run with 2-fs time step at 293 K under the harmonic restraint for atoms in a peptide with a force constant of 10.0 kcal∙mol^−1^ Å^−2^. In this manner, only the hydration structure was sampled during the production run. At first, production runs of 10 ns were conducted for the MD-LP and MD-noLP simulations of the GEKG peptide, and it was confirmed that a production run of 2 ns was long enough for the convergence of the solvent density maps (Supplementary Note 1 and Figs S1–S4). Therefore, production runs of 2 ns were conducted for all other peptides. In all of the production runs, the coordinates of all atoms were saved at every 0.1 ps.

Finally, the hydration structures were visualized in the form of solvent density maps for oxygen and hydrogen atoms in the hydration water molecules. These maps were calculated from the MD trajectory in the last 1 ns of a production run by counting the number of electrons in water molecules visiting the 0.05 Å cube voxels that were defined to divide the simulation system^59^. A high solvent density means that the water molecules frequently visit and/or reside in that voxel. In the density maps, the distance and angular distributions of water oxygen atoms are quantitatively described by tracing the solvent density in the volume elements in a polar coordinate system (*r*, *θ*, *φ*) fixed to each targeted atom (Fig. 1B). The maps displayed in all figures were calculated using 0.25 Å cube voxels. Table 1 provides the quantitative characteristics of the density distributions of water oxygen atoms around each polar group, such as their peak positions, half width at half maximum (HWHM) of the peak profile, and the total amount of hydrating water molecules.

To calculate the interaction potential between the peptides and a single water molecule, we first putted the water oxygen atom in the center of each 0.05-Å cube voxel. Then the water molecule was rotated in every possible orientation by fixing the position of the water oxygen atom, and in each orientation the non-bond potential (the sum of the electrostatic and Lennard-Jones potentials) between the peptide and the water molecule was calculated. The minimum value of the non-bonded potential among all possible orientation was defined as the interaction potential between the peptide and the water molecule at the position of the voxel.

### MD simulations of proteins

The MD-LP and MD-noLP simulations were performed for Sec7 (the accession code of the PDB: 4A4P), RNase-Sa (4J5G^34^) and HK (2C2A^35^) to calculate hydration structures of these proteins. The atomic models of Sec. 7, RNase and HK were immersed in a water box with the dimensions of 106 × 90 × 114 (containing 28,900 water molecules), 90 × 114 × 90 (24,800) and 126 × 126 × 100 Å^3^ (43,800), respectively. Under periodic boundary conditions, the dimensions were wide enough to separate each molecule from its periodic images by more than 40 Å.

The procedure of equilibration MD runs for these proteins is the same with that for the peptides. After the equilibration at 1 atm, the dimensions of the systems for Sec. 7, RNase-Sa and HK shrank to 100 × 84 × 108, 84 × 108 × 84 and 120 × 120 × 94 Å^3^, respectively. Production runs of 10 ns were conducted under the harmonic restraint for atoms in proteins with a force constant of 10.0 kcal∙mol^−1^∙Å^−2^. The coordinates of all atoms were saved at every 1 ps.

The solvent density maps for oxygen and hydrogen atoms in the hydration water molecules around these proteins were calculated from the 1–2 ns MD trajectories with the 0.5 Å cube voxels as done in the analyses for the peptides. It was confirmed that the convergence of the hydration structure was obtained within the first 2-ns of a production run. The calculated hydration structures were compared with the locations of the crystal water sites found around the proteins.

## Electronic supplementary material

Supplementary Information
