# Supplementary material for: Influences of lone-pair electrons on directionality of hydrogen bonds formed by hydrophilic amino acid side chains in molecular dynamics simulation
Source: Sci Rep. 2017 Nov 20;7:15859. doi: 10.1038/s41598-017-16203-w (PMC5696464; doi:10.1038/s41598-017-16203-w)
Supplement: Supplementary file 1 — Supplementary Information [file 41598_2017_16203_MOESM1_ESM.pdf]

Supplementary Information for

# "Influences of lone-pair electrons on directionality of hydrogen bonds formed by hydrophilic amino acids in molecular dynamics simulation"

Tomotaka Oroguchi [a,b,*] and Masayoshi Nakasako [a,b]

[a]Department of Physics, Faculty of Science and Technology, Keio University,

3-14-1 Hiyoshi, Kohoku-ku, Yokohama, 223-8522 Japan

[b]RIKEN SPring-8 Center, 1-1-1 Kohto, Sayo, Sayo-gun, Hyogo 679-5148 Japan

[*] To whom correspondence should be addressed. Phone: +81-45-566-1703. Fax: +81-45-566-1672.

E-mail: oroguchi@phys.keio.ac.jp.

**Supplementary Note 1: Convergence of hydration structure**

The width of the time-window, in which the solvent density maps calculated from a MD trajectory, was first investigated for a 10-ns production run of both the MD-LP and MD-noLP simulations for the Gly-Glu-Lys-Gly (GEKG) peptide. Supplementary Figure S1 compares the solvent density maps of oxygen atoms in hydration water molecules around the peptide calculated for the time-windows of 1.0-1.2, 1.0-1.5 and 1.0-3.0 ns in the production run with that calculated from the EHDF. The map from the time-window of 1.0-1.2 ns displayed noise in the bulk region, indicating the insufficient window width. In the map of the time-window of 1.0-1.5 ns, the noise level was significantly reduced. The time-window of 1.0-3.0 ns gave the solvent density map with the noise densities at very small levels. The map was suitable to compare with that from the EHDF. Supplementary Figure S2 shows the solvent density maps of oxygen and hydrogen atoms of hydration water molecules around the OE1 and OE2 atoms calculated for the time-windows of 1.0-1.2, 1.0-1.5, 1.0-2.0 and 1.0-3.0 ns. As well as the maps in Figure S1, wider time-window gave maps with lower noise levels both in the density maps and in the profiles of distance and angular distributions. These tendencies were common between the MD-LP and MD-noLP simulations. Therefore, we set the size of the time-window at 1 ns in this study.

Next, we examined whether the solvent densities calculated by using the 1-ns time-window gave almost the same maps at any moment in the 10-ns trajectory. Supplementary Figure S3 compares the solvent density maps calculated in the time-intervals of 1-2, 4-5 and 9-10 ns in the trajectory. The maps were almost independent of the three calculations. Around the OE1 and OE2 atoms of glutamate side chain, the solvent density distributions calculated in the time-intervals of 1-2, 4-5 and 9-10 ns were also equivalent with each other (Supplementary Fig. S4). This tendency was common in both the MD-LP and MD-noLP simulations. Therefore, these results allowed us to calculate the solvent density distributions for the last 1 ns in a 2-ns production run of peptides for comparisons with the EHDFs.

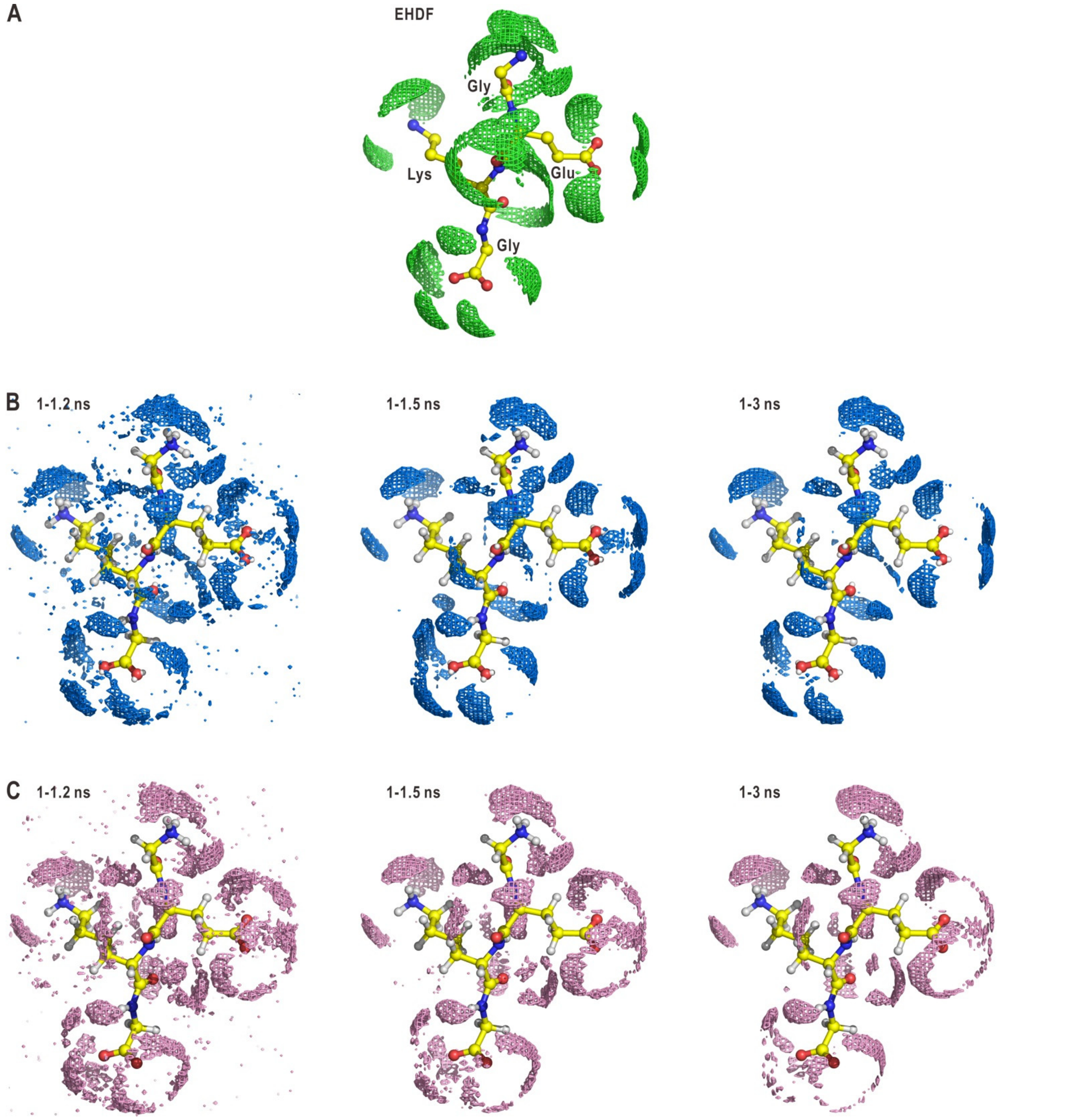


**Supplementary Figure S1.** (A) Solvent density map of oxygen atoms in hydration water molecules around the GEKG peptide predicted by using the EHDFs. Solvent density maps obtained from the 1-1.2,

1-1.5, and 1-3 ns trajectories of the MD-LP (B) and MD-noLP (C) simulations of 10 ns. The density maps are contoured at 1.6 e·$Å^{-3}$.

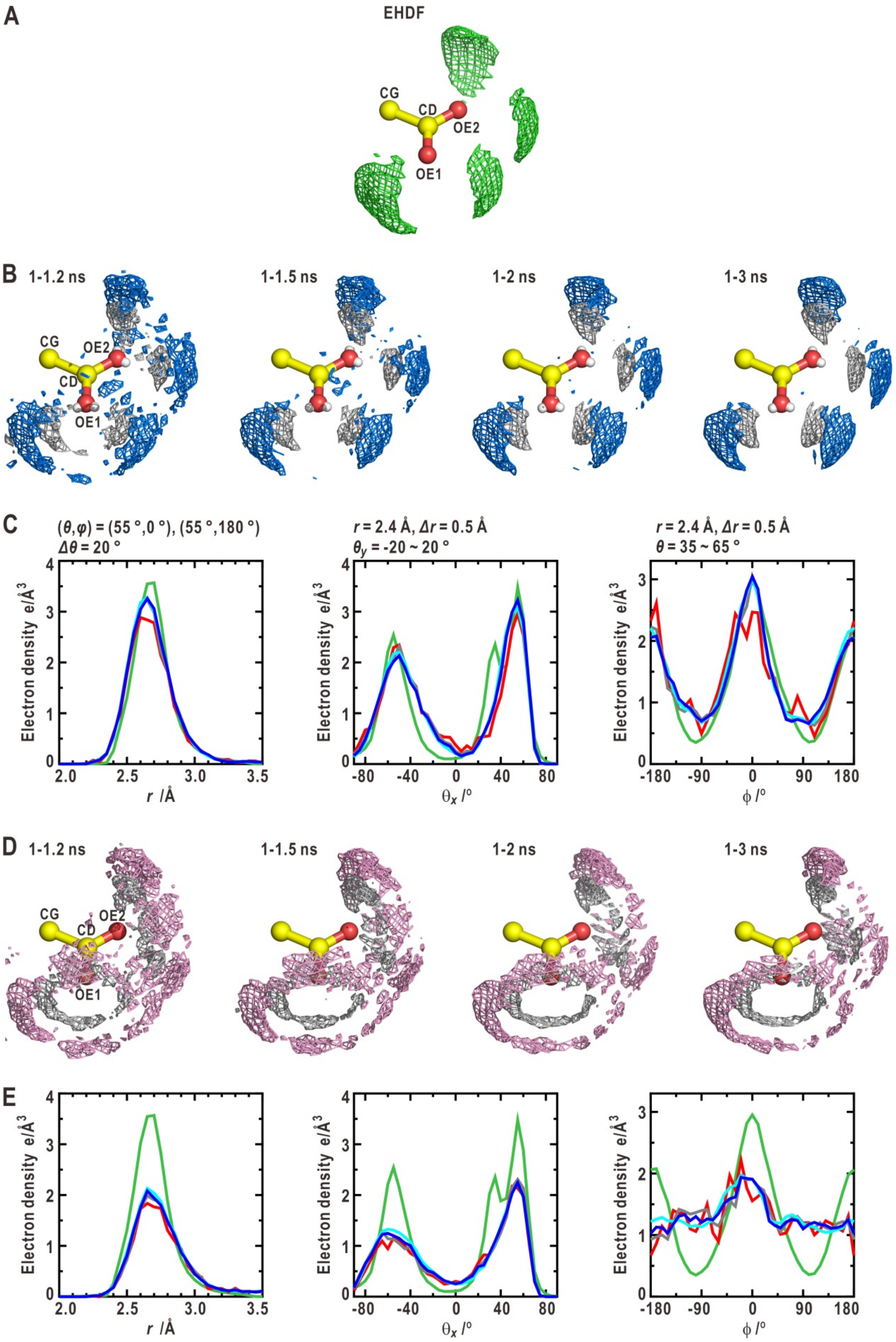
A
EHDF
CG
CD
OE2
OE1
B
1-1.2 ns
1-1.5 ns
1-2 ns
1-3 ns
C
(θ,φ) = (55 °,0 °), (55 °,180 °)
Δθ = 20 °
r = 2.4 Å, Δr = 0.5 Å
θy = -20 ~ 20 °
r = 2.4 Å, Δr = 0.5 Å
θ = 35 ~ 65 °
Electron density e/Å3
r /Å
θx /°
ϕ /°
D
1-1.2 ns
1-1.5 ns
1-2 ns
1-3 ns
E
Electron density e/Å3
r /Å
θx /°
ϕ /°

**Supplementary Figure S2.** (A) Solvent density maps, which display the distribution of oxygen and/or hydrogen atoms in hydration water molecules, around the OE1 and OE2 atoms in the side chain of glutamate in the GEKG peptide predicted by using the EHDFs. (B) Solvent density maps calculated from the 1-1.2, 1-1.5, 1-2, and 1-3 ns trajectories of the 10-ns MD-LP simulation. The positions of the LP charge sites used in the MD-LP simulation are indicated by the small white spheres. The density maps for oxygen atoms in the EHDF (green), MD-LP (blue) and MD-noLP (pink) simulations, and for hydrogen atoms (white) are contoured at 1.6 and 0.4 e·Å$^{-3}$, respectively. These schemes are used in the subsequent illustrations of solvent density maps in the Supplementary Information. (C) The profiles of distance (left), $\theta_x$ (center) and $\varphi$ (right) distributions of oxygen atoms in hydration water molecules in the MD-LP simulation. The distance distribution is calculated by averaging those in the four clusters of the solvent densities. In addition, the two angular distributions are averaged for the solvent densities around the OE1 and OE2 atoms. The parameters necessary for these calculations in the coordinate system shown in Fig. 1B of the main text are labelled at the top of each plot. The scheme for plotting the profiles is also used in the subsequent illustrations in the Supplementary Information. Profile of the EHDF (green) is compared with those calculated from the 1.0-1.2 (red), 1.0-1.5 (grey), 1.0-2.0 (blue), and 1.0-3.0 (cyan) ns trajectories. Solvent density maps (D) and distribution profiles (E) calculated from the 1-1.2, 1-1.5, 1-2, and 1-3 ns trajectories of the 10-ns MD-noLP simulation.

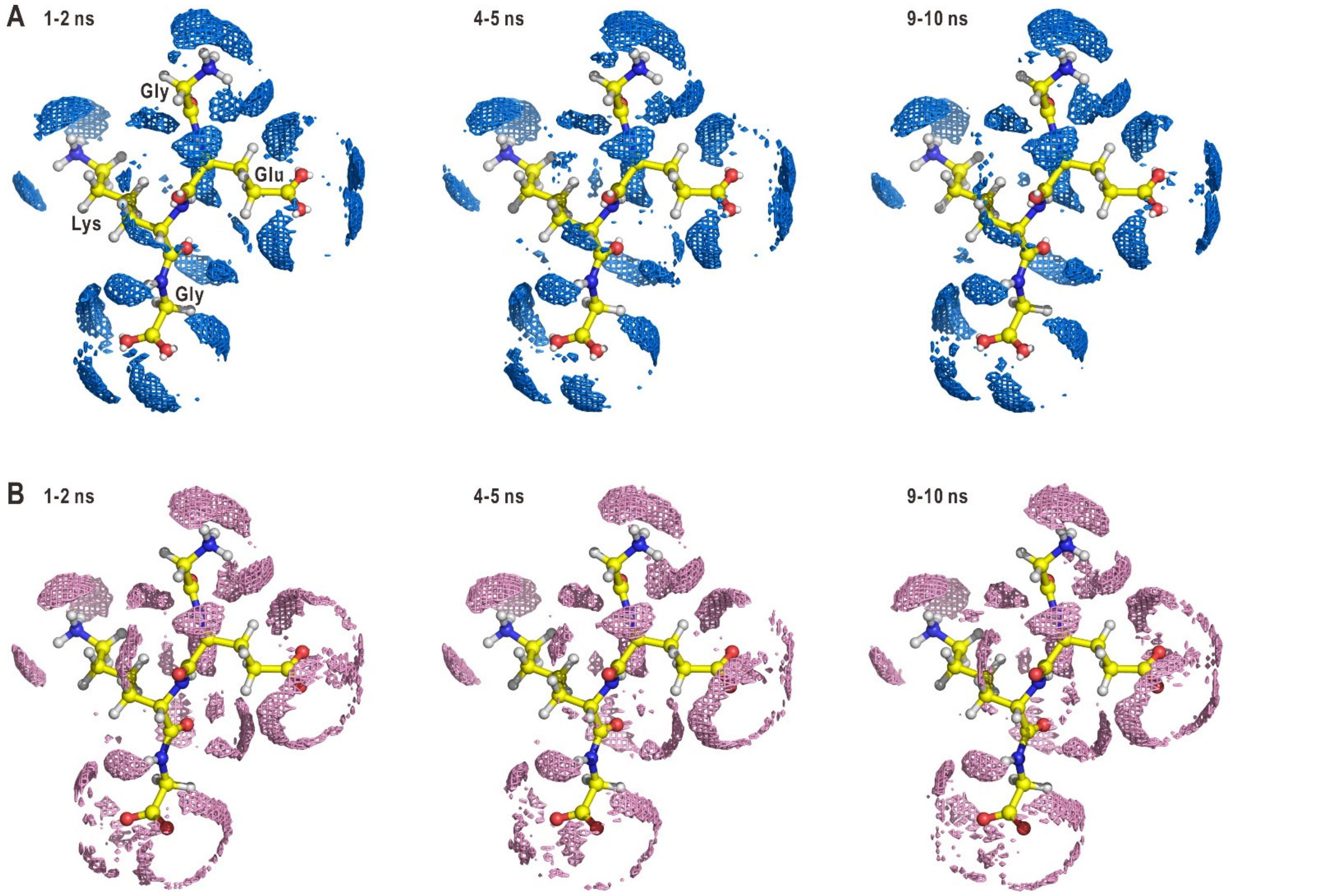


**Supplementary Figure S3.** Solvent density maps of oxygen atoms in hydration water molecules around the GEKG peptide obtained from the 1-2, 4-5, and 9-10 ns trajectories of the MD-LP (A) and MD-noLP (B) simulations of 10 ns.

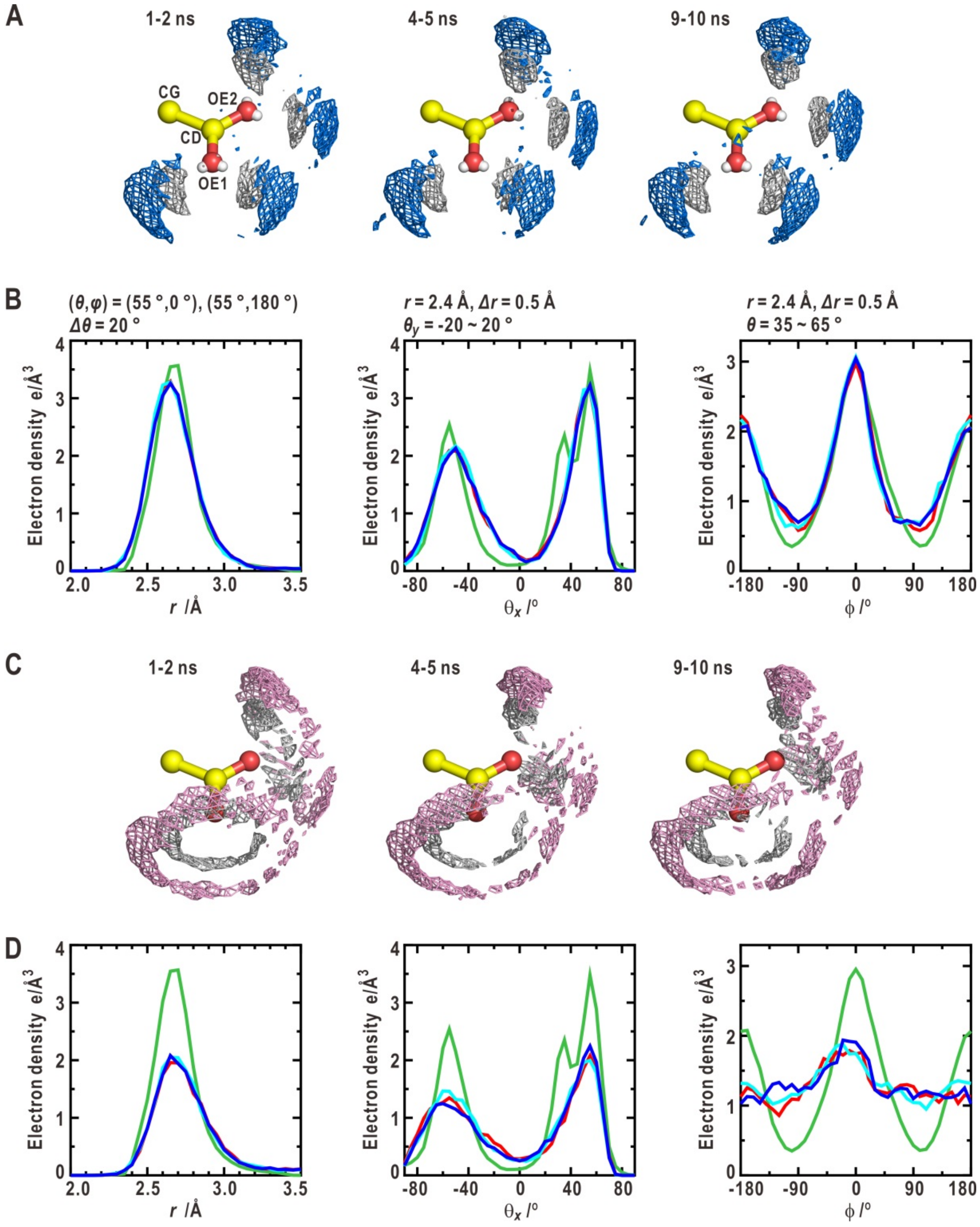


**Supplementary Figure S4.** Solvent density maps and the distribution profiles around the OE1 and OE2 atoms in the side chain of glutamate calculated from the 1-2, 4-5 and 9-10 ns time-intervals for the 10-ns MD-LP (panels (A) and (B)) and MD-noLP (panels (C) and (D)) simulations. The distance (left), $\theta_x$ (center), and $\varphi$ (right) distributions of oxygen atoms in hydration water molecules were averaged around the OE1 and OE2 atoms. The profile of the EHDF (green) are compared with those from the 1-2 (blue),

4-5 (red) and 9-10 (cyan) ns intervals.

**Supplementary Note 2: Hydration structures around the deprotonated $sp^2$-hybridized oxygen atoms in aspartate**

Supplementary Figure S5 shows the solvent density distribution around the deprotonated $sp^2$-hybridized OD1 and OD2 atoms in the side chain of the aspartate. The MD-LP simulation reproduced the distribution of oxygen atoms of hydration water molecules in the EHDF regarding the profiles and peak positions in the distance and angular distributions (Supplementary Fig. S5C and Table 1). The simulation gave four density peaks of the oxygen atoms of the hydration water molecules. The center of each of these peaks was located in the directions of OD1(OD2)-LP (($\theta_x, \varphi$) = (+55°, 0°) and (−55°, 180°)). The density peak of the hydrogen atoms of water molecules appeared between the OD1 (OD2) atom and the density peak of water oxygen atoms, indicating the directionality of the H-bond.

The MD-noLP simulation gave a solvent density distribution different from that of the MD-LP simulation and the EHDF. The oxygen atoms of the hydration water molecules around the OD1 (OD2) atom were distributed in toroidal shape, the center of which was located in the CG-OD1 (CG-OD2) direction, and had little prominent peaks in the $\varphi$ distribution (Supplementary Fig. S5C and Table 1). The densities of the hydration water molecules residing around the OD1 (OD2) atom were smaller than those in the MD-LP simulation, suggesting that the interaction between OD1 (OD2) and hydration water molecules is weaker than that in the MD-LP simulation. Between the oxygen atoms of the hydration water molecules and OD1 (OD2) atoms, the hydrogen atoms of the hydration water molecules were distributed in an arc shape.

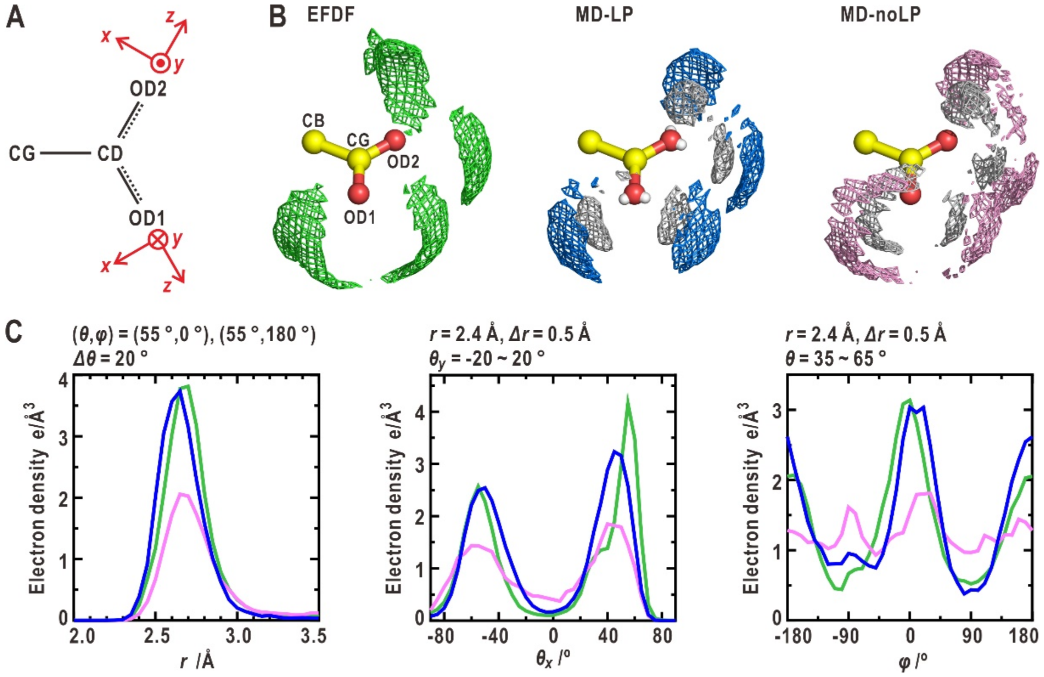


**Supplementary Figure S5.** (A) The coordinate systems used for describing the solvent density distribution around the OD1 and OD2 atoms in the side chain of aspartate. (B) Comparison of the solvent density maps around the OD1 and OD2 atoms among the EHDF, and MD-LP and MD-noLP simulations for the Gly-Asp-Lys-Gly (GDKG) peptide. (C) The profiles of the distance (left panel), $\theta_x$ (center), and $\varphi$ (right) distributions of oxygen atoms in hydration water molecules. The distance distribution is calculated by averaging the four clusters of the solvent densities of the oxygen atoms. The $\theta_x$- and $\varphi$-distributions are averaged over the solvent densities around the OD1 and OD2 atoms. The green, blue, and pink lines are the profiles from the EHDF, MD-LP, and MD-noLP simulations, respectively. The coloring for plotting the profiles is also used in the subsequent illustrations in the Supplementary Information.

**Supplementary Note 3: Hydration structure around the side chain of histidine in the HID state**

Around the protonated ND1 atom, the MD-LP and MD-noLP simulations gave the distribution of oxygen atoms of hydration water molecules similar to that of the EHDF with respect to the peak positions and profiles in the angular ($\theta_x$ and $\theta_y$) distributions (Supplementary Fig. S6 and Table 1). However, the densities of the hydration water molecules residing around the ND1 atom were smaller than those in the EHDF (Supplementary Fig. S6C). The peak positions and profiles of the distance distributions in the simulations were inconsistent with those in the EHDF. For simulating better the EHDF regarding the profiles in the distance and angular distributions, more attractive interactions between the hydration water molecules and the N-H group would be necessary

Regarding the deprotonated $sp^2$-hybridized NE2 atom, the MD-LP simulation simulated well the angular distribution in the EHDF (Supplementary Fig. S6D). In contrast, the distance distribution of the solvent density differed from that of the EHDF regarding the peak position (2.65 Å in the MD-LP, and 2.75 Å in the EHDF) and the profile. When the NE2-LP charge pair has less attractive interactions with hydration water molecules in the simulation, the differences would become small. In the MD-noLP simulation, the hydration water molecules around the NE2 atom were distributed in a wide range of $\theta_y$ with two broad maxima at $\theta_y = \pm 80°$, while the $\theta_y$-distribution of the EHDF and the MD-LP simulation had single peaks at $\theta_y = 0°$. This finding suggests that the LP set at the NE2 atom in the MD-LP simulation is necessary to localize the hydration water molecules within the plane of the imidazole ring.

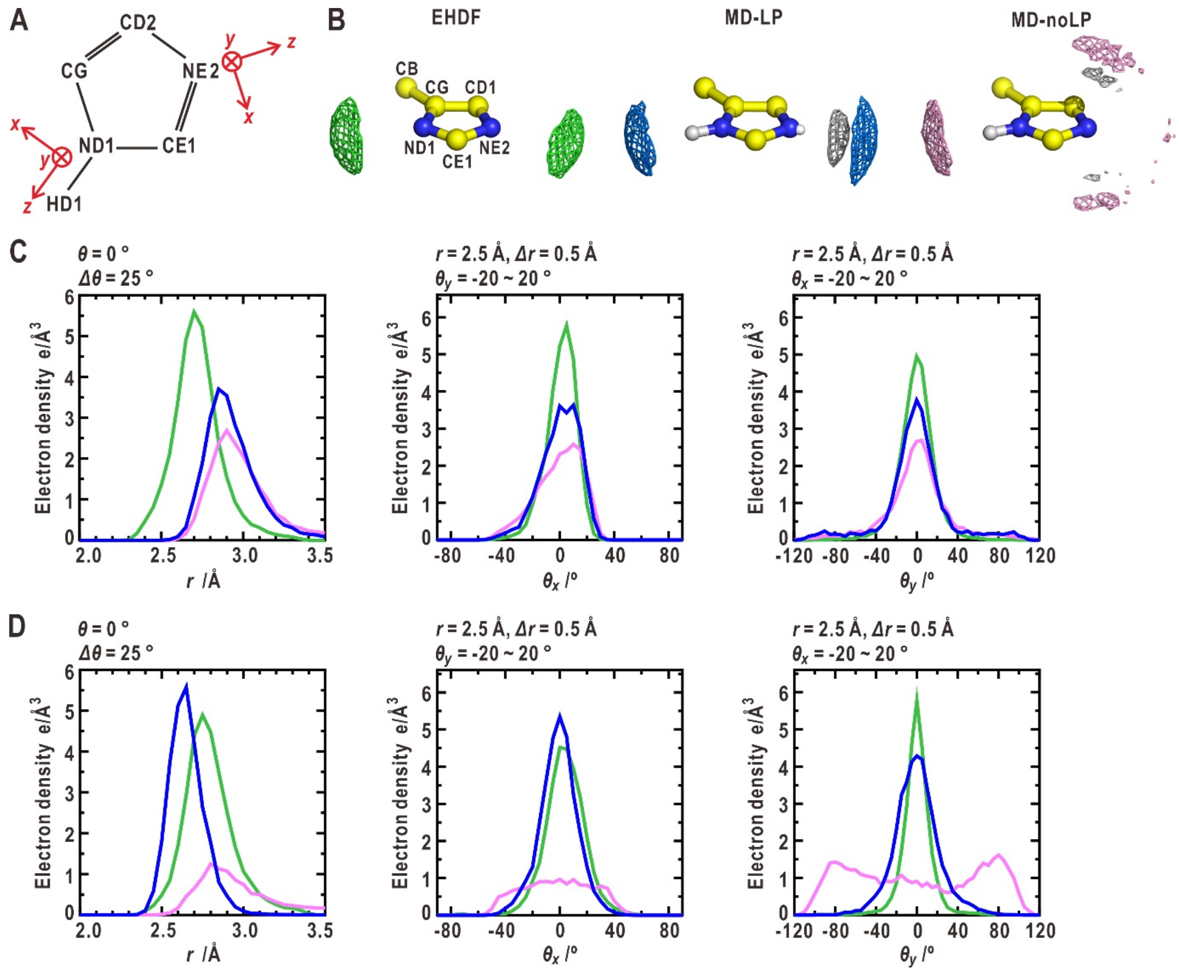


**Supplementary Figure S6.** (A) The coordinate systems used for describing the solvent density distributions around the ND1 and NE2 atoms in the imidazole ring of histidine. (B) Comparison of the solvent density maps around the imidazole ring of histidine among the EHDF, and MD-LP and MD-noLP simulations for the Gly-His (HID state)-Gly tripeptide. The profiles of distance (left panel), $\theta_x$ (center), and $\theta_y$ (right) distributions of the water oxygen densities around the ND1 and NE1 atoms are plotted in panels (C) and (D), respectively.

**Supplementary Note 4: Hydration structures around the protonated oxygen atoms in the *sp*$^3$-hybridization in the side chains of serine and threonine**

In the EHDFs around the OG atom of serine (Supplementary Fig. S7B) and the OG1 atom of threonine (Supplementary Fig. S8B), the solvent densities are distributed in semi-toroidal shapes with three prominent peaks at $\varphi = 0°$ and $\pm 90°$. These peaks are attributed to the three configurations of the CB-OG (CB-OG1) bond (Supplementary Figs. S7C-E and S8C-E). Therefore, MD simulations were conducted for the three configurations. The solvent densities were summed over the simulations and normalized so that the total sum of the densities within the toroid was equal to that in the EHDFs. In the MD-LP simulation, two LPs on the side opposite to the OG-HG (OG1-HG1) bond were modeled by assuming that the OG (OG1) atom of serine (threonine) is in the *sp*$^3$-hybridization.

The MD-LP and MD-noLP simulations for each configuration gave almost the same solvent density distributions both around serine (Supplementary Fig. S7C-E) and threonine (Supplementary Fig. S8C-E). They were composed of two major peaks with narrow widths and another with a broad maximum (Table 1). For the configuration in which the dihedral angle of CA-CB-OG(OG1)-HG(HG1) was 0°, a major peak appeared in the direction of $\varphi = 0°$. A broad minor peak in the opposite direction of the major peak ($-180° < \varphi < -90°$, $90° < \varphi < 180°$) was caused by the frequent residence of the hydration water molecules in a narrow pocket formed between the side and main chains. Since the peak was absent from the EHDFs and independent of the two MD simulations, the van der Waals attractions between the hydration water molecules and the atoms forming the pocket were predominant, rather than the dipole-dipole interactions between the hydration water molecules and the OG(OG1)-LPs.

For the configuration, in which the dihedral angle was ±90°, the major density peak appeared at $\varphi = \pm 90°$, identical to those found in the EHDFs of serine and threonine. The minor peak located on the opposite side of the OG-HG (OG1-HG1) bond was smaller than that observed in the case where the dihedral angle was 0°. The hydration water molecules comprising the minor peaks had contacts with the

atoms forming the main chain. Consequently, the dipole-dipole interactions with the OG-LPs would have little influence on their residence in the region of the minor peaks, because the shape and size of the minor peaks were independent of the MD-LP and MD-noLP simulations.

In both serine and threonine, the summed solvent densities resembled that of the EHDFs with respect to the profiles including the peak positions, except for the influences from the minor peaks (Supplementary Figs. S7F-G and S8F-G, and Table 1). It is also noteworthy that the minor differences in the distributions from the MD-LP and MD-noLP simulations was consistent with the present results for tyrosine (Fig. 4) and also with the other theoretical studies using QM calculations[1,2]. These results and the analysis for the tyrosine side chain indicate that the LP electrons exert a minor influence on the hydration structures around the protonated $sp^3$-hybridized oxygen atoms.

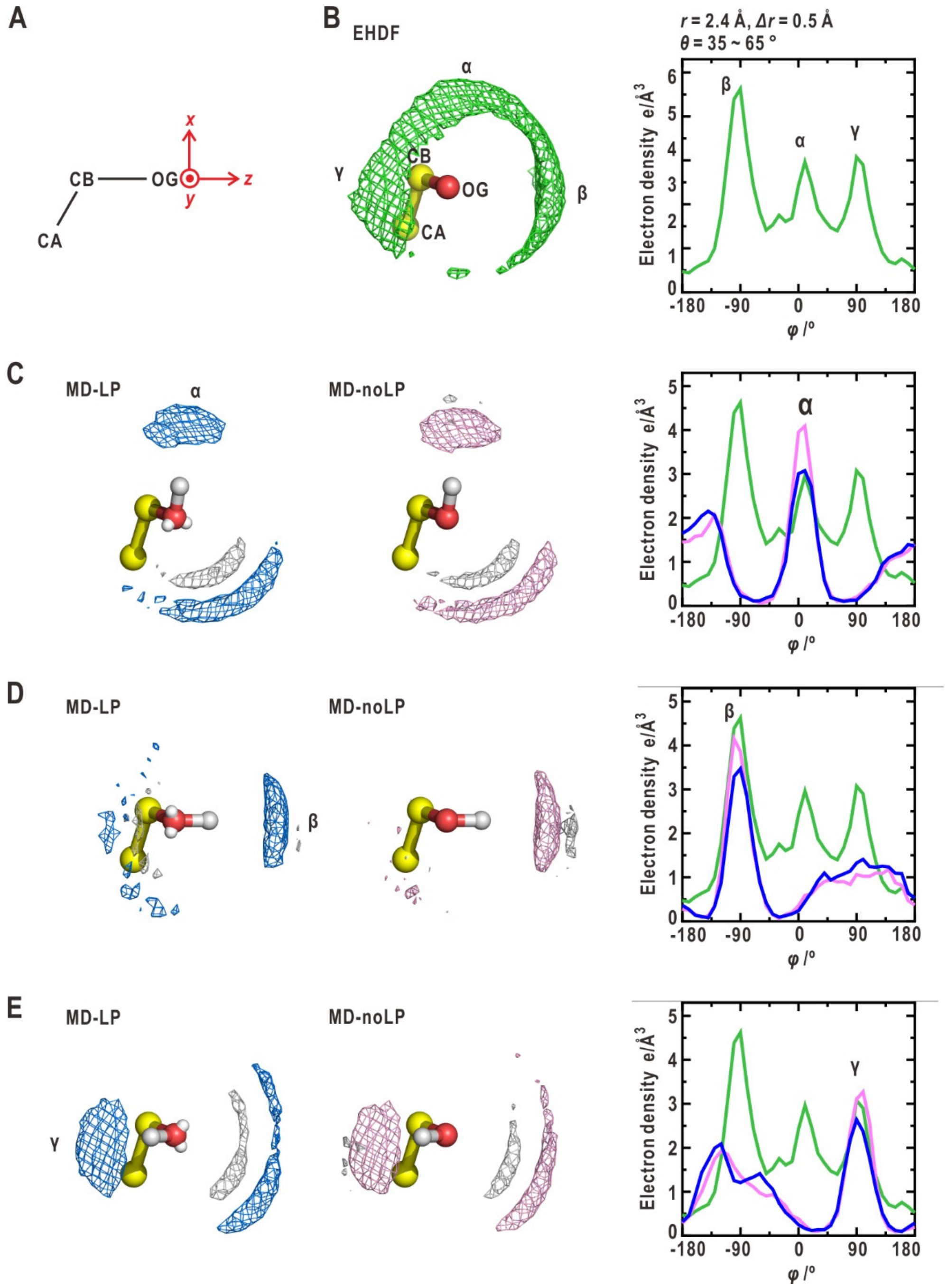
A
x
CB
OG
z
y
CA
B
EHDF
α
γ
CB
OG
CA
β
r = 2.4 Å, Δr = 0.5 Å
θ = 35 ~ 65 °
β
α
γ
Electron density e/Å3
φ /°
-180 -90 0 90 180
C
MD-LP
α
MD-noLP
α
Electron density e/Å3
φ /°
D
MD-LP
β
MD-noLP
β
Electron density e/Å3
φ /°
E
MD-LP
γ
MD-noLP
γ
Electron density e/Å3
φ /°

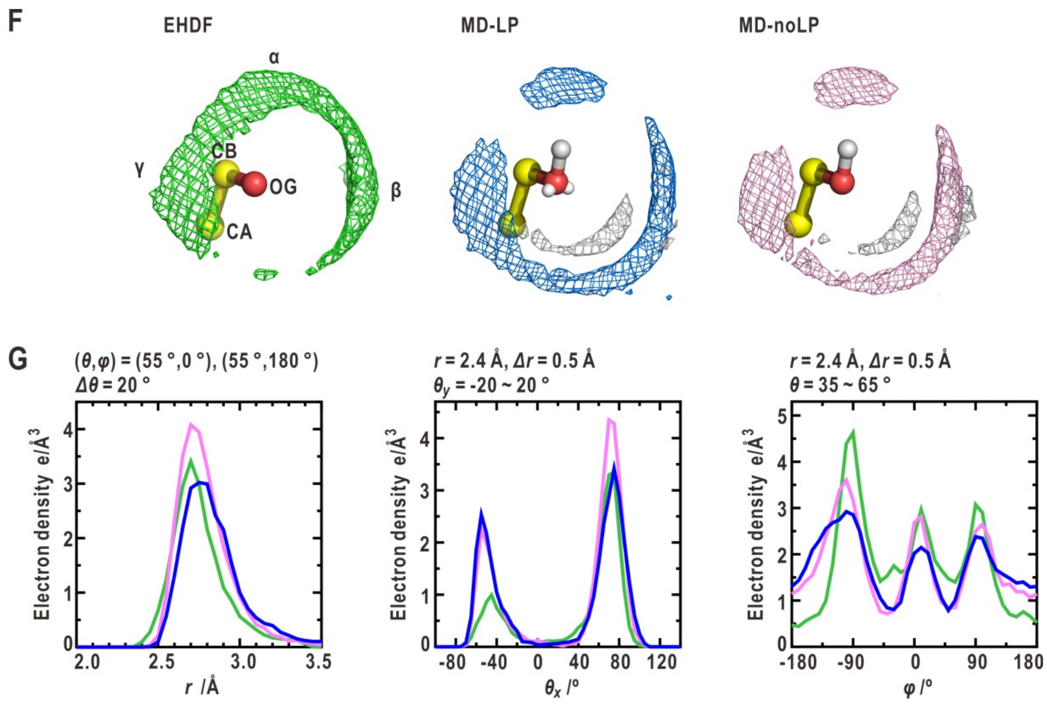


**Supplementary Figure S7.** (A) The coordinate system used for describing the solvent density distributions around the OG atom in the side chain of serine. (B) The solvent density map around the OG atom in the EHDF (left panel), and the $\varphi$ distribution (right). The three prominent peaks in the $\varphi$ distribution are labeled as α, β, and γ. The solvent density distributions in the configurations of CA-CB-OG-HG at the torsion angles of –180° (C), –90° (D), and +90° (E) are compared between the MD-LP and MD-noLP simulations. The profiles of $\varphi$-distributions of oxygen atoms in hydration water molecules in the MD-LP (blue line) and MD-noLP (pink) simulations are plotted and compared with that of the EHDF (green) in the right panels. (F) Comparison of the solvent density map of the EHDF with those of oxygen atoms in hydration water molecules summed over the three configurations in the MD-LP and MD-noLP simulations. (G) The profiles of distance (left), $\theta_x$ (center), and $\varphi$ (right) distributions of solvent densities of the EHDF, summed densities of oxygen atoms in hydration water molecules in the MD-LP and MD-noLP simulations.

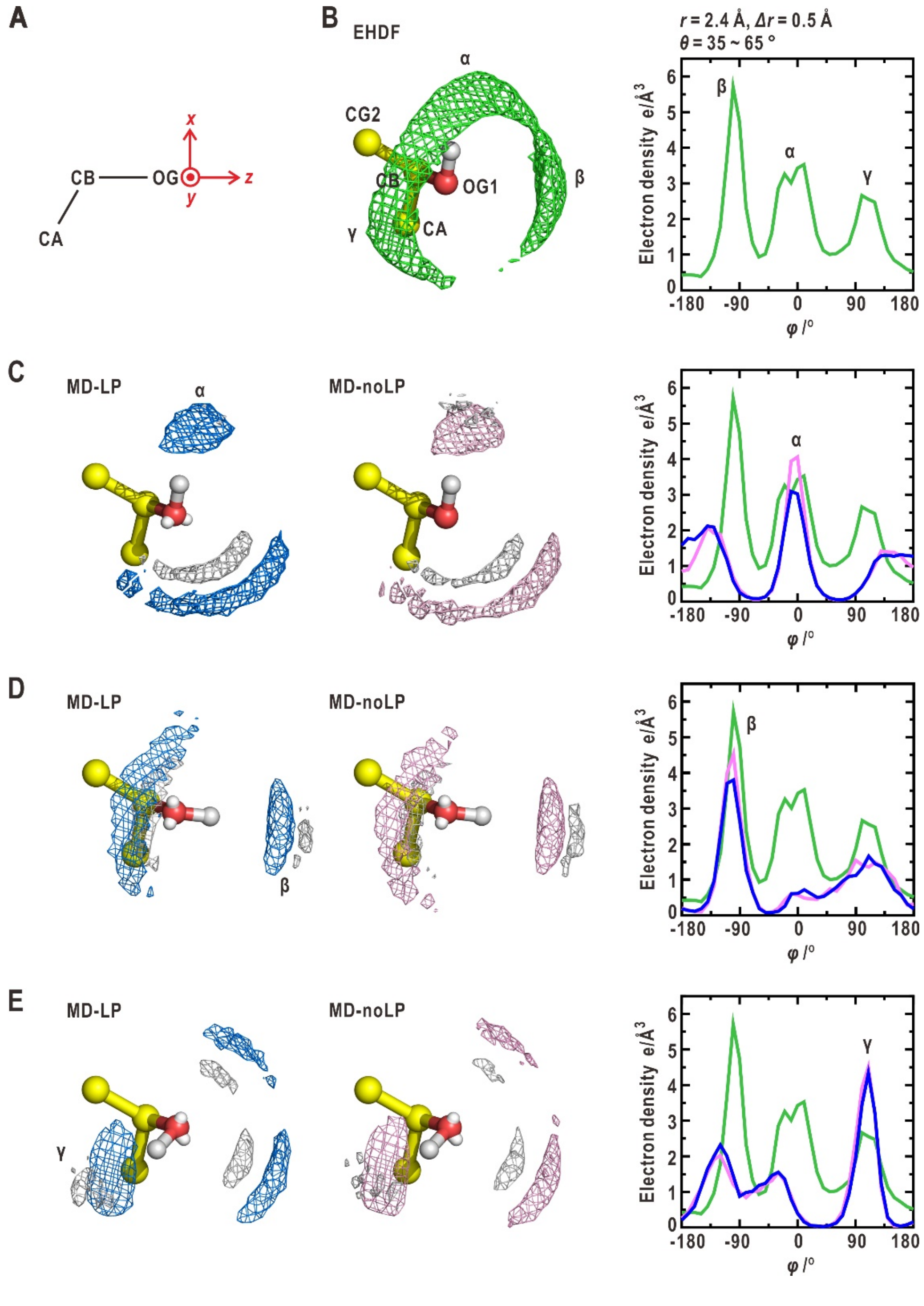
A
CB
OG
CA
x
y
z
B
EHDF
α
β
γ
CG2
CB
OG1
CA
r = 2.4 Å, Δr = 0.5 Å
θ = 35 ~ 65 °
Electron density e/Å3
φ /°
-180
-90
0
90
180
C
MD-LP
MD-noLP
D
MD-LP
MD-noLP
E
MD-LP
MD-noLP

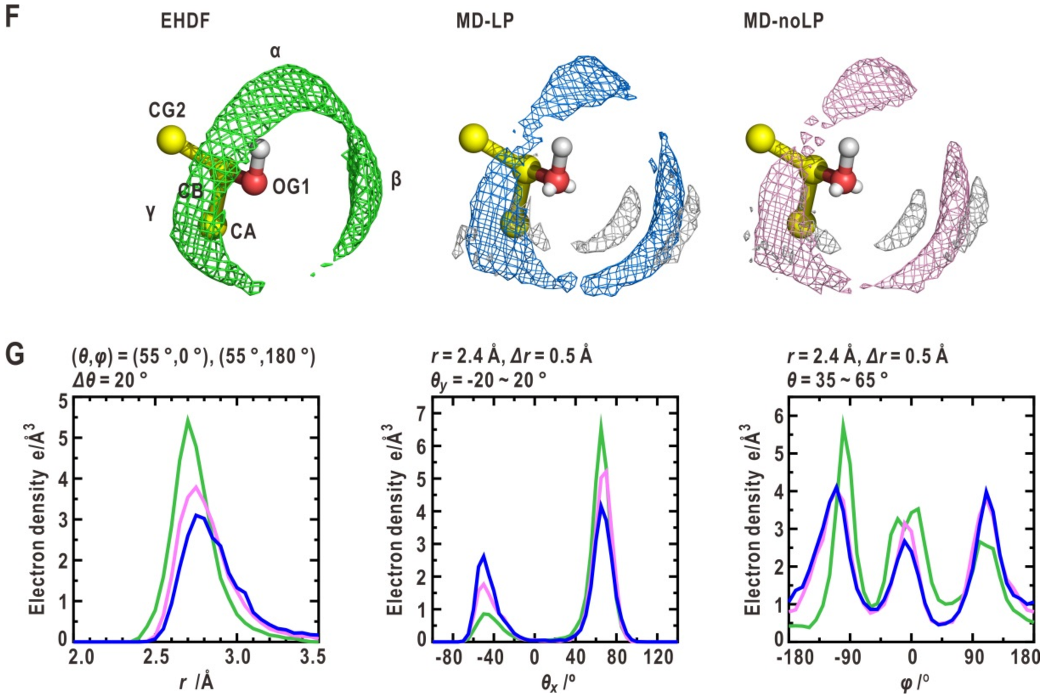


**Supplementary Figure S8.** (A) The coordinate system used for describing the solvent density distributions around the OG1 atom in the side chain of threonine. (B) The solvent density map around the OG1 atom in the EHDF (left panel), and the $\varphi$ distribution (right). The three prominent peaks in the $\varphi$ distribution are labeled as α, β, and γ. The solvent density distributions in the configurations of CA-CB-OG1-HG1 at the torsion angles of –180° (C), –90° (D), and +90° (E) are compared between the MD-LP and MD-noLP simulations. The profiles of $\varphi$-distributions of oxygen atoms in hydration water molecules in the MD-LP (blue line) and MD-noLP (pink) simulations are plotted and compared with that of the EHDF (green) in the right panels. (F) Comparison of the solvent density map of the EHDF with those of oxygen atoms in hydration water molecules summed over the three configurations in the MD-LP and MD-noLP simulations. (G) The profiles of distance (left), $\theta_x$ (center), and $\varphi$ (right) distributions of solvent densities of the EHDF, summed densities of oxygen atoms in hydration water molecules in the MD-LP and MD-noLP simulations.

**Supplementary Note 5: Hydration structure around the protonated oxygen atom in the *sp*$^2$-hybridization in the side chain of tyrosine**

The OH atom in the side chain of tyrosine is typically assumed to be *sp*$^3$-hybridized. However, this atom sometimes appears to be *sp*$^2$-hybridized in the crystal structures of proteins[3,4]. Therefore, in this study, the hydration structure around the OH atom in the *sp*$^2$-hybridization was also calculated with the LP charge site determined as described in the Method section of the main text.

The LP charge site of the OH atom was placed on the opposite side of the HH atom. In both the MD-LP and MD-noLP simulations, the solvent densities predominantly appeared in the direction of the OH-HH bond (Supplementary Fig. S9). The density maps resembled those observed in the simulations for the *sp*$^3$-hybridized OH atom with respect to the distance and angular distributions (Supplementary Figs. S9C-D and Table 1).

On the OH-LP side, a single density peak of oxygen atoms in water molecules appeared in the MD-LP simulation. While this density peak resembled those existing on the OH-HH side in terms of the profiles and the peak positions in the distribution (Table 1), the densities of the hydration water molecules forming the density peaks were smaller than those of the OH-HH side (Supplementary Figs. S9D-E). The hydrogen atoms of hydration water molecules were localized between the oxygen atoms of water molecules and the OH atom of tyrosine, indicating the directionality of the H-bond. The peak position in the distance distribution suggests that the interactions between the hydration water molecules and the OH-LP group are quite weak.

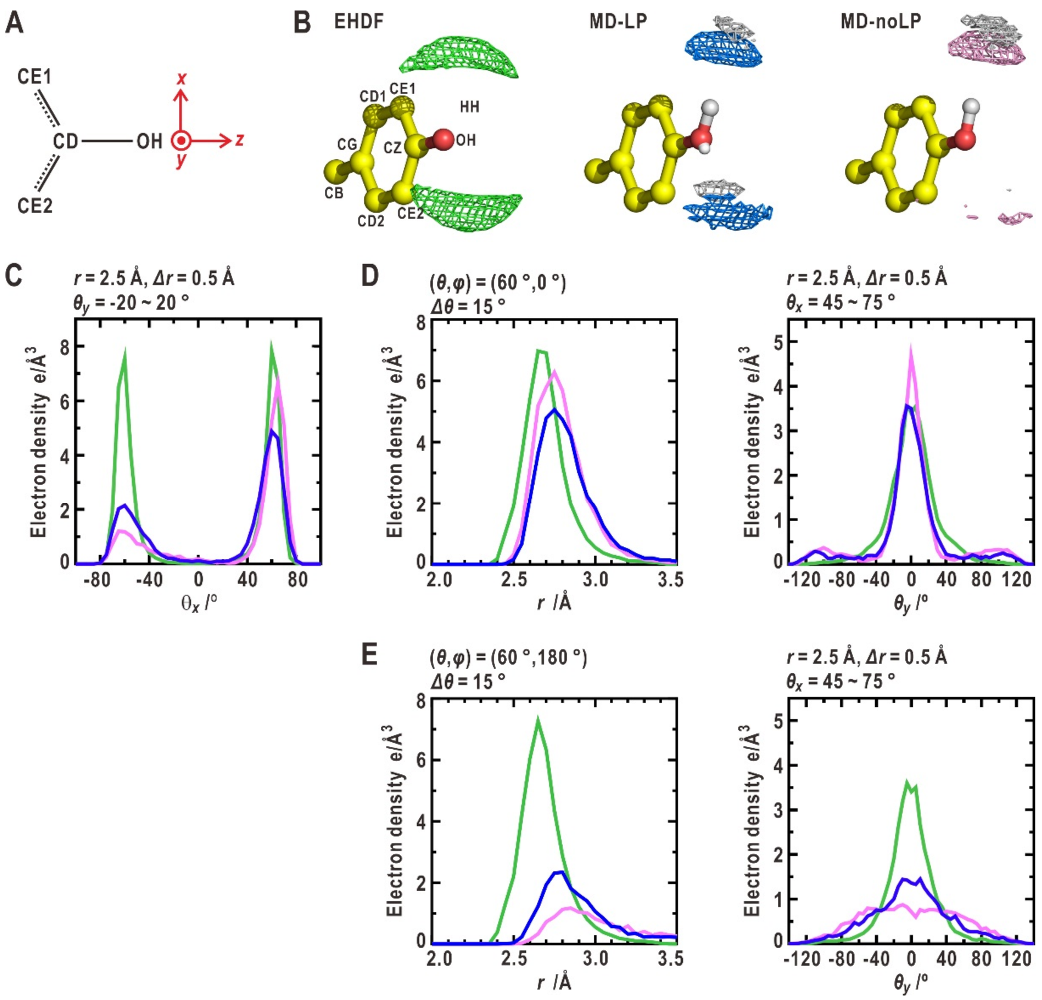


**Supplementary Figure S9.** (A) The coordinate system used for describing the solvent density distributions around the OH atom in the side chain of tyrosine. (B) Comparison of the solvent density maps around the $sp^2$-hybridized OH atom among the EHDF, MD-LP and MD-noLP simulations. (C) The $\theta_x$ distribution of water oxygen densities around the protonated OH atom. The distance and $\theta_y$ distributions on the (D) OH-HH bond side of the protonated OH atom and (E) OH-LP side.

**Supplementary Note 6: Hydration structure around the protonated nitrogen atom of the side chain of tryptophan**

The NE1 atom in the side chain of tryptophan is protonated under physiological conditions. Both the MD-LP and MD-noLP simulations reproduced the hydration structures observed in the EHDF (Supplementary Fig. S10) of tryptophan, as well as the case for lysine, and arginine. In all these hydration structures, the single density peak of water oxygen atoms appeared in the direction of the NE1-HE1 bond ($\theta_x = \theta_y = 0°$ in the $\theta_x$ and $\theta_y$ distributions). However, the N atom was less hydrated in these simulations compared to that found in the EHDF (Table 1). One likely reason for this difference might be the small polarization of the NE1-HE1 group determined by the RESP procedure and under the normalization condition of the EHDF, in which one water molecule is supposed to form a hydrogen bond with this group. These points should be addressed in future studies.

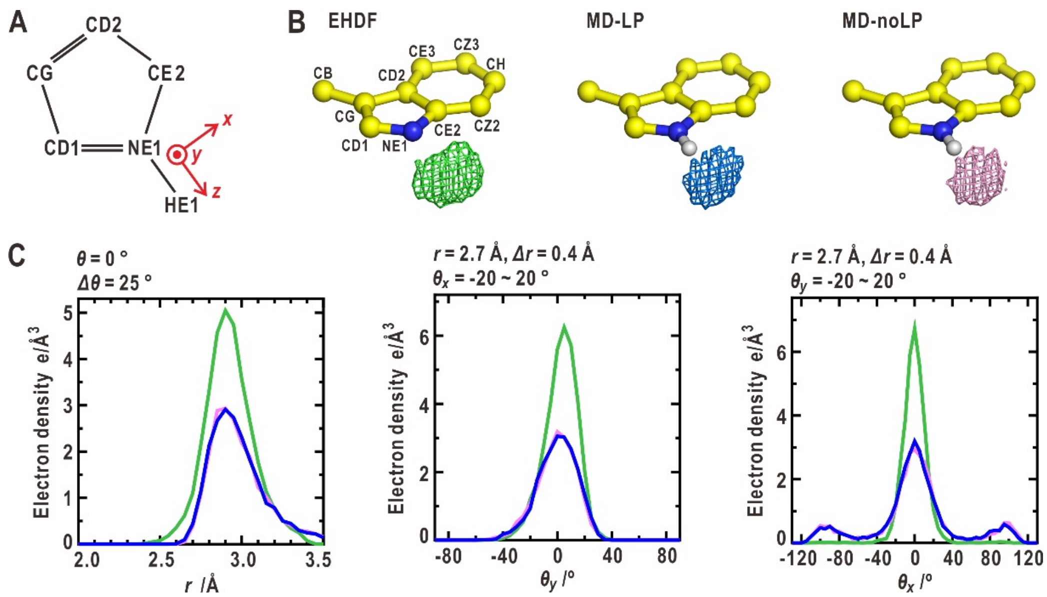


**Supplementary Figure S10.** (A) The coordinate system used for describing the solvent density distributions around the NE1 atom in the side chain of tryptophan. (B) Comparison of the solvent density maps around the NE1 atom among the EHDF, and MD-LP and MD-noLP simulations. (C) The profiles of distance (left panel), $\theta_x$ (center), and $\theta_y$ (right) distributions of water oxygen densities around the protonated NE1 atom.

**Supplementary Note 7: EHDF for glutamine and asparagine**

The hydration structures around the side chains of glutamine and asparagine, which include both deprotonated $sp^2$-hybridized oxygen atoms and protonated nitrogen atoms, have not been studied in this work. As reported previously[7], the tips of the side chains in a number of glutamine and asparagine residues in the crystal structures are incorrectly modeled with incorrect torsion angles, even though the structures are refined at resolutions better than 2.2 Å.

The EHDFs for the side chains of these two amino acids are unsuitable for use as references for the MD simulations, and therefore the validation on the geometrical characteristics in the H-bonds formed by the side chains of asparagine and glutamine was difficult at the present time. However, the incorporation of LP charge sites into a force field was possible. The tentatively determined charge values on the protonated nitrogen atom and deprotonated oxygen atom both in their $sp^2$-hybridization were listed in Table 1.

**Supplementary Note 8: Comparison of the solvent density distributions from the MD simulations using the charge sets determined by the present and the standard RESP procedures**

By using the standard RESP procedure[6,7], we also determined the charges for glutamate and lysine In the first step, the two structures with the conformations of α-helix and β-strand were constructed for a dipeptide, in which each amino acid was blocked by the acetyl and *N*-methyl groups. The rotamer states of the side chain in each conformation were set to the parameters reported by the literature[7]. Subsequently, the QM-ESPs calculated for those conformations were used to determine the charges of the targeted amino acid by using the multi-conformational RESP fitting with the restraint scheme described in the main text. As well as our RESP procedure applied for the GEKG peptide, the charges were determined for both of the models with and without LP sites. The resultant charge sets were implemented in the AMBER force field, and then the hydration structures around the GEKG peptide were calculated by conducting MD-LP and md-noLP simulations for 2 ns.

The charges on the side chains of glutamine and lysine determined using the present and standard RESP procedures were nearly the same (Supplementary Figs. S11-S13 and Table S4). In both MD-LP and MD-noLP simulations, the solvent density distributions around these side chains were almost the same between the two RESP procedures (Supplementary Figs. S12 and S13). Although two RESP procedures resulted in different charges values on the peptide bonds (Supplementary Table S4), the hydration structures around the main chain were similar (Supplementary Fig. S11). Based on the results described above, we decided to employ the present RESP procedure for the determination of charges in the tri- or tetrapeptides.

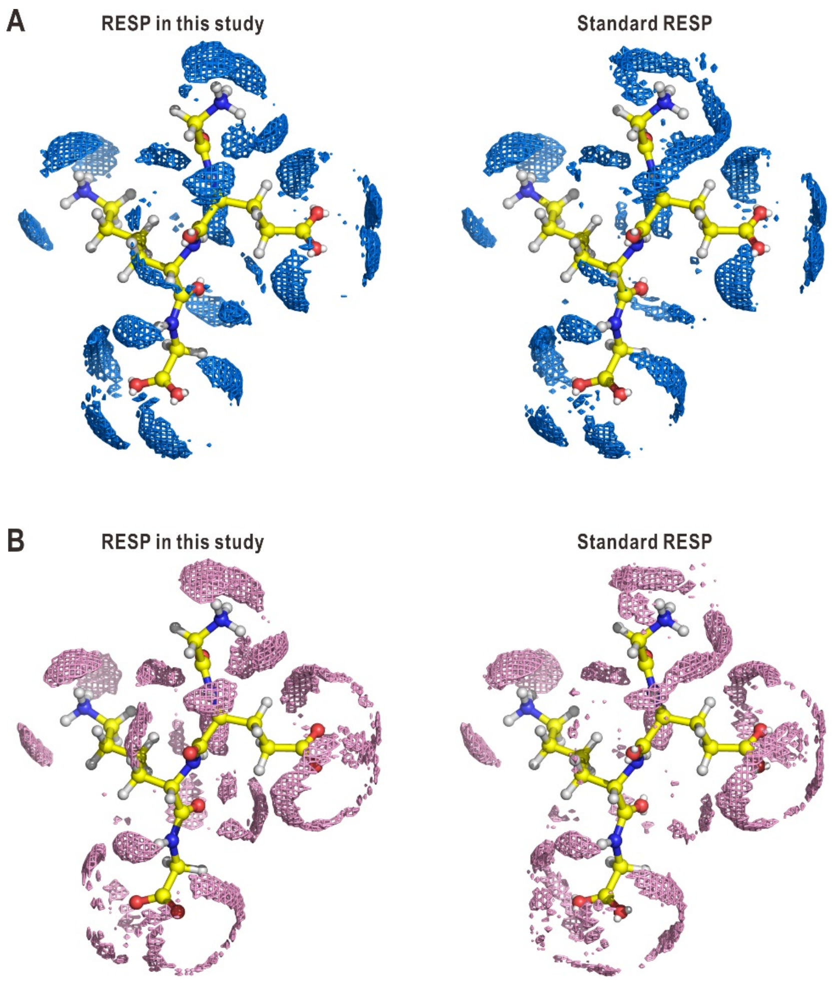


**Supplementary Figure S11.** (A) Solvent density maps of oxygen atoms in hydration water molecules around the GEKG peptide from the MD-LP simulations using the charges determined by the present (left) and the standard (right) RESP procedures. (B) Solvent density maps from the MD-noLP simulations using the charges determined by the present (left) and the standard (right) RESP procedures.

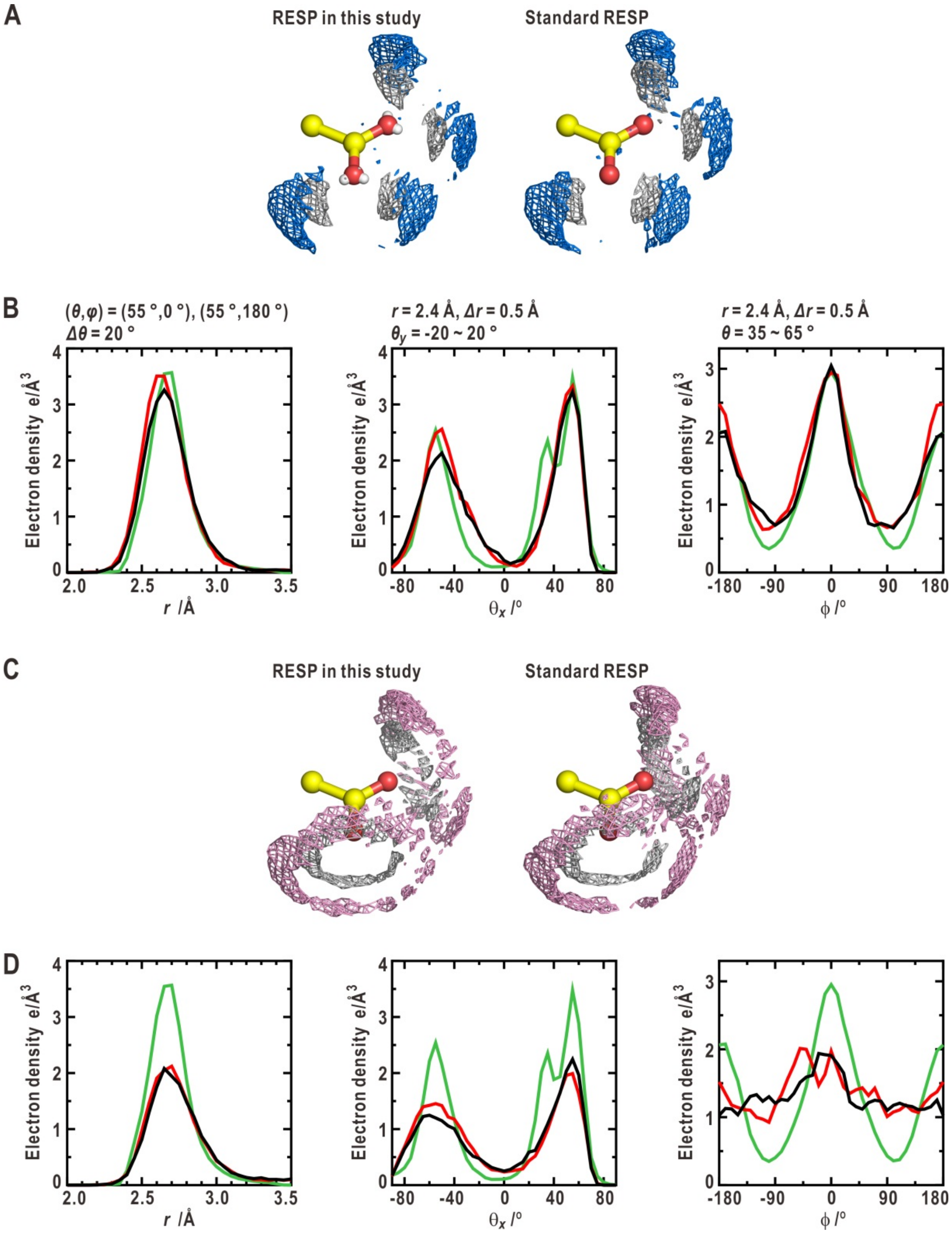


**Supplementary Figure S12.** (A) Solvent density maps around the OE1 and OE2 atoms in the side chain of glutamate in the GEKG peptide from the MD-LP simulations using the charges determined by the present (left) and the standard (right) RESP procedures. (B) Profiles of the distance (left),$\theta_x$ (center), and $\varphi$ (right) distributions of oxygen atoms in hydration water molecules around the OE1 and OE2 atoms in

the EHDF (green line), the MD-LP simulations using the charges determined by the present (black) and the standard (red) RESP procedures. The distance distribution is calculated by averaging those in the four clusters of the solvent densities. In addition, the $\theta_x$ and $\varphi$ distributions are averaged for the solvent densities around the OE1 and OE2 atoms. (C) Solvent density maps around the side chain of glutamate from the MD-noLP simulations using the charges determined by the present (left) and the standard (right) RESP procedures. (D) Profiles of the distance (left), $\theta_x$ (center), and $\varphi$ (right) distributions of solvent densities around the OE1 and OE2 atoms in the MD-noLP simulations. The coloring scheme of the lines are the same with that in panel (B).

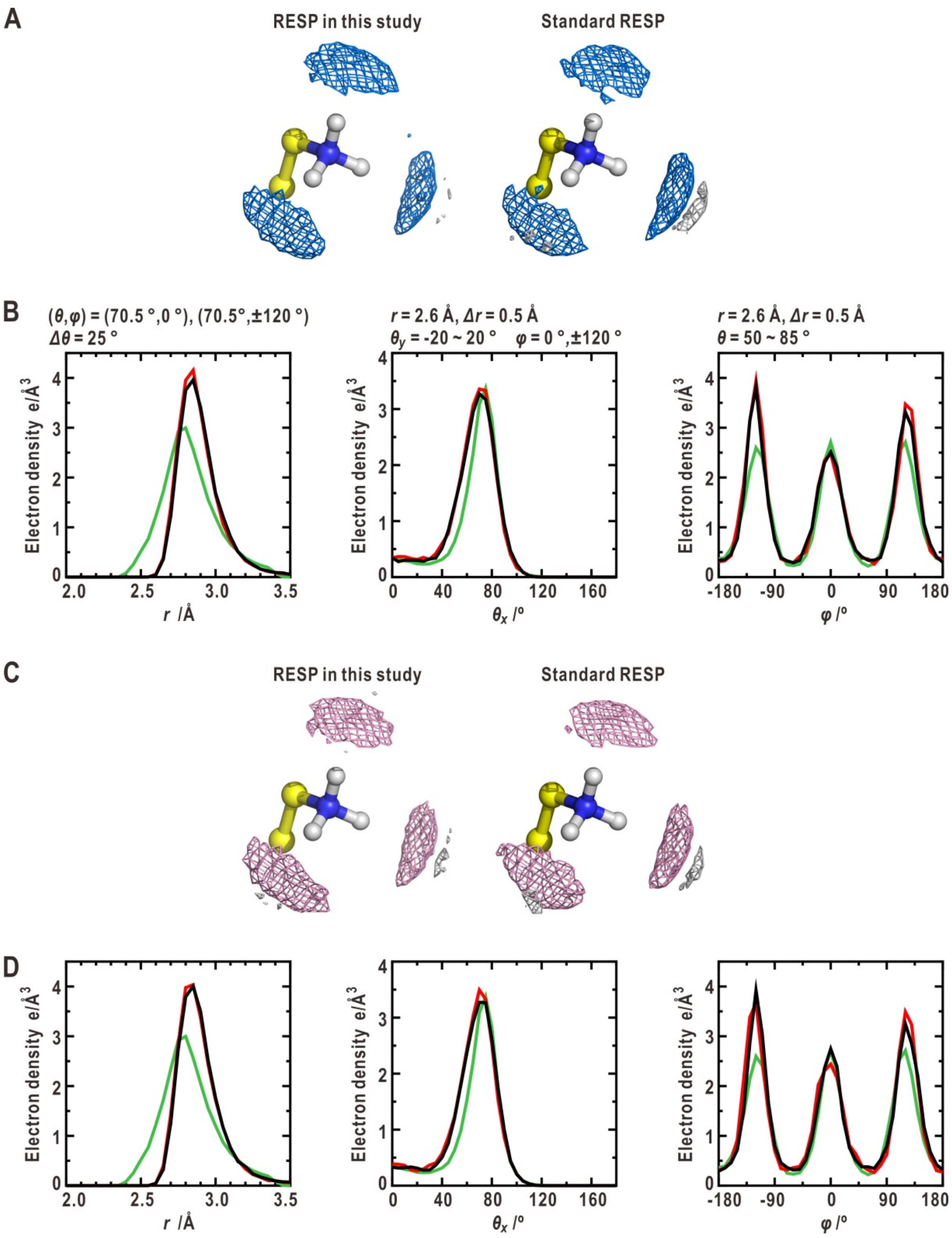


**Supplementary Figure S13.** (A) Solvent density maps around the $NH_3^+$ group in the side chain of lysine in the GEKG peptide from the MD-LP simulations using the charges determined by the present (left) and the standard (right) RESP procedures. (B) Profiles of distance (left panel), $\theta_x$ (center), and $\varphi$ (right) distributions of oxygen atoms in hydration water molecules around the $NH_3^+$ group calculated

from the EHDF (green line), the MD-LP simulations using the charges determined by the present (black) and the standard (red) RESP procedures. The coordinate system used for calculating the profiles is shown in Figs 1B and 5A of the main text. (C) Solvent density maps from the MD-noLP simulations using the charges determined by the present (left) and the standard (right) RESP procedures. (D) Profiles of the distance (left panel), $\theta_x$ (center), and $\varphi$ (right) distributions of the oxygen atoms in hydration water molecules around the $NH_3^+$ group in the MD-noLP simulations.

**Supplementary Note 9: Influence of water models on the solvent density distribution in the MD-LP and MD-noLP simulations**

The solvent densities in the EHDFs around the side chains of glutamate and lysine in the Gly-Glu-Lys-Gly peptide were compared among the MD-LP and MD-noLP simulations using the TIP3P[8] (Figure 1), TIP4P/Ew[9], TIP5P[10], and SPC/E[11] models (Supplementary Figs S14 and S15). While the solvent density distributions around the $NH_3^+$ group in the side chain of lysine were similar to the EHDF in all the simulations, those around the deprotonated oxygen atoms in the $sp^2$-hybridization in the side chain of glutamate depended on the models of water molecules as well as the two types of MD simulations.

In the MD-LP simulations using the TIP3P (Fig. 1), SPC/E (Supplementary Fig. S15A-B), and TIP4P/Ew (Supplementary Fig. S15C-D)) models, the distributions of oxygen atoms in hydration water molecules around the side chains of the glutamate residues resembled that of the EHDF with regard to the profiles and peak positions in the distance and angular distributions (Supplementary Table S5). Hydrogen atoms of hydration water molecules were localized along the OE1 (OE2)-LP directions between the OE1 (OE2) atom and the oxygen atoms of hydration water molecules. In the MD-LP simulations, the TIP3P model provided hydration structures that displayed the highest consistency with the EHDF among the three models.

In the MD-noLP simulations using the TIP3P (Fig. 1), SPC/E (Supplementary Fig. S15A-B), and TIP4P/Ew (Supplementary Fig. S15C-D) models, the distributions of oxygen atoms in hydration water molecules were inconsistent with those from the MD-LP simulations and in the EHDF. The solvent densities were distributed in toroidal shapes around the OE1 (OE2) atom at approximately $\theta = 55°$. In addition, the low density in the distance distribution suggested that the OE1 (OE2) atoms were less hydrated in the MD-noLP simulations than in the MD-LP simulations.

Both the MD-LP and MD-noLP simulations using the TIP5P model gave the distribution of the

oxygen atoms in hydration water molecules inconsistent with the EHDF (Supplementary Figs. S15E-F and Table S5). The solvent densities distributed in toroidal shapes around the OE1 (OE2) atoms resembled those from the MD-noLP simulations using the TIP3P, TIP4P/Ew, and SPC models rather than those from the MD-LP simulations using the three models (Supplementary Fig. S15F). The densities of the hydration water molecules in the toroidal shapes were significantly smaller than those in the MD-noLP simulations for the other models of water molecules.

Regarding the computational cost for MD simulations, the TIP4P/Ew model increases drastically the degrees of freedom of the simulation systems. In addition, the SPC/E model uses different configuration parameters to those obtained experimentally. For these reasons, the TIP3P model was selected as the model for water molecule in this simulation study.

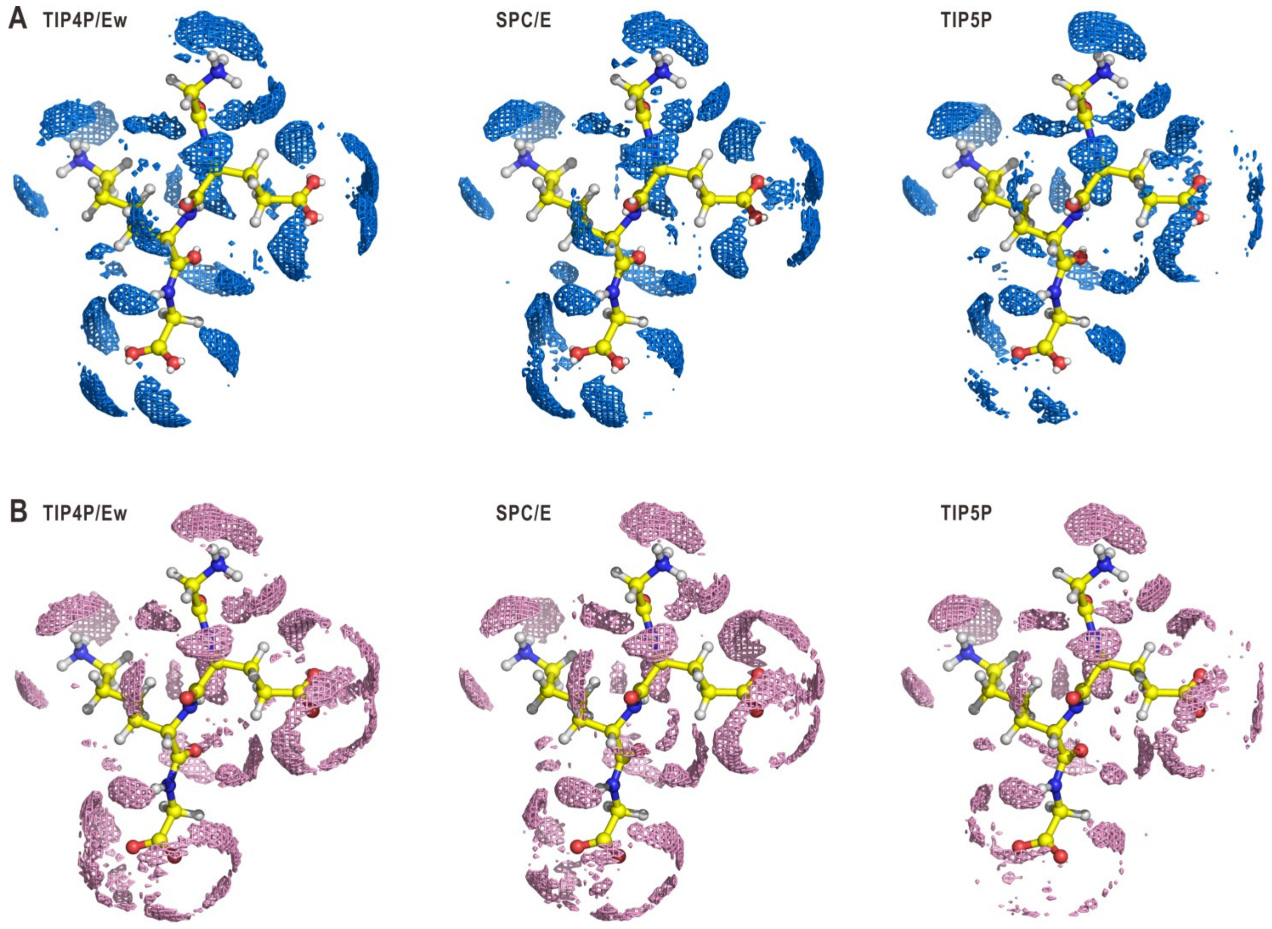


**Supplementary Figure S14.** Solvent density maps of water oxygen atoms from the MD-LP (A) and MD-noLP (B) simulations using the TIP4P/Ew, SPC/E, and TIP5P models.

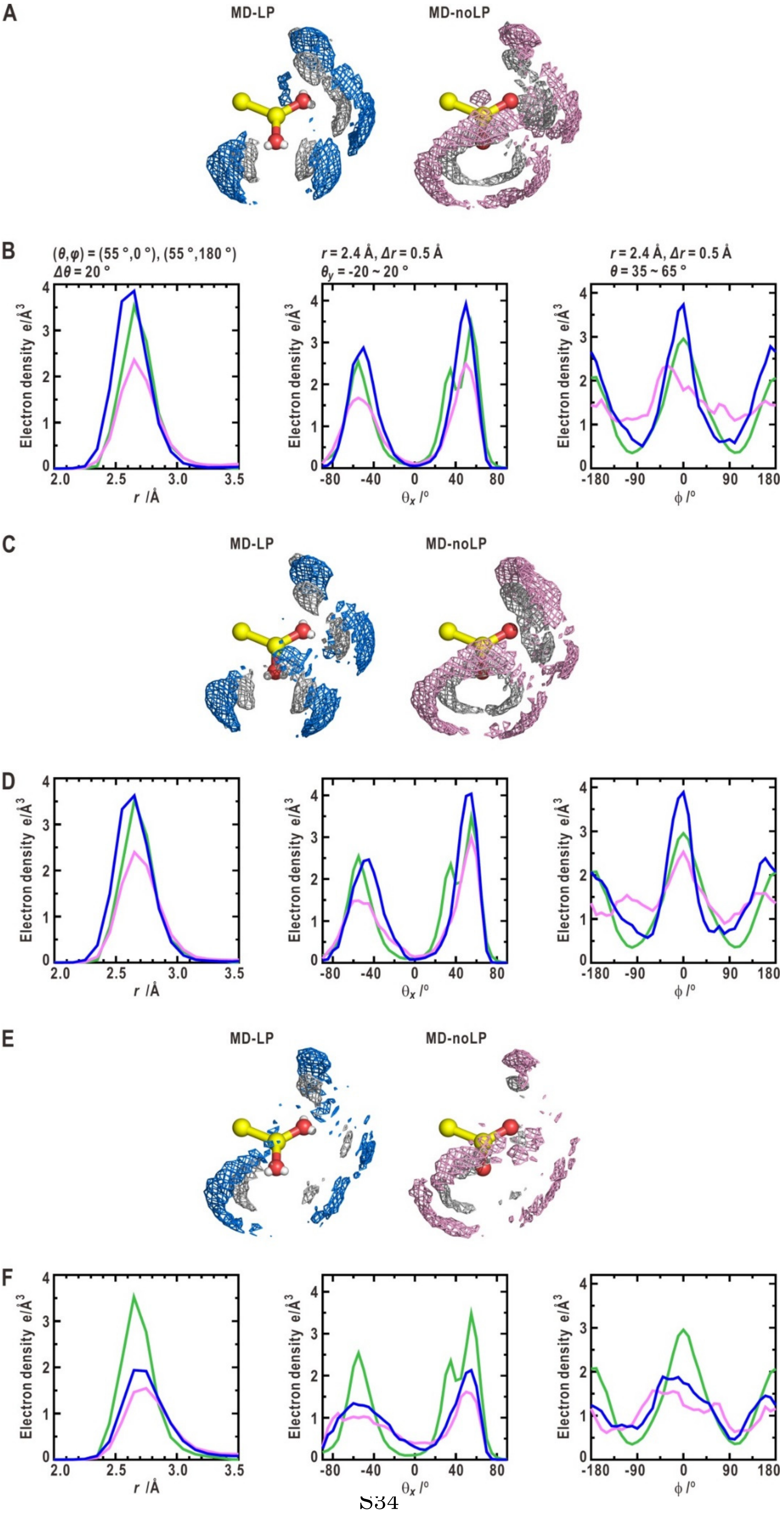
A
MD-LP
MD-noLP
B
(θ,φ) = (55 °,0 °), (55 °,180 °)
Δθ = 20 °
r = 2.4 Å, Δr = 0.5 Å
θy = -20 ~ 20 °
r = 2.4 Å, Δr = 0.5 Å
θ = 35 ~ 65 °
Electron density e/Å3
r /Å
θx /°
ϕ /°
C
MD-LP
MD-noLP
D
Electron density e/Å3
r /Å
θx /°
ϕ /°
E
MD-LP
MD-noLP
F
Electron density e/Å3
r /Å
θx /°
ϕ /°

**Supplementary Figure S15.** Solvent density maps around the OE1 and OE2 atoms in the side chain of glutamate in the GEKG peptide immersed in water bath composed of SPC/E (panels (A) and (B)), TIP4P/Ew ((C) and (D)), and TIP5P ((E) and (F)). Panels (A) illustrate solvent density maps of the MD-LP and MD-noLP simulations. Panels (C) and (E) compare the solvent density maps only from the MD-LP and MD-noLP simulations. Plots in panels (B), (D) and (F) are profiles of the distance (left), $\theta_x$ (center), and $\varphi$ (right) distributions, respectively. The distributions are averaged for the OE1 and OE2 atoms.

**Supplementary Table S1.** Atom-centered and off-atom charges of amino acids determined by the RESP protocol applied to the non-blocked tri- and tetrapeptides. In this study, even though the hydration structures calculated from the MD simulations are not shown for glutamine and asparagine owing to the reasons described in the Methods section in the main text, it is possible to perform calculations for these amino acids. The determined charges are also listed in this table.

| **Without LP electrons** | | **With LP electrons** | |
|---|---|---|---|
| **Charge site** | **Charges** | **Charge site** | **Charges** |
| | **Glutamate** | | |
| N | -0.860084 | N | -0.897270 |
| H | 0.412786 | H | 0.444355 |
| CA | 0.092112 | CA | 0.110056 |
| HA | 0.121019 | HA | 0.133800 |
| CB | -0.035896 | CB | -0.067795 |
| HB2 | 0.054904 | HB2 | 0.059574 |
| HB3 | 0.054904 | HB3 | 0.059574 |
| CG | -0.218415 | CG | -0.032361 |
| HG2 | 0.037065 | HG2 | 0.027754 |
| HG3 | 0.037065 | HG3 | 0.027754 |
| CD | 0.853802 | CD | 0.282909 |
| OE1 | -0.812340 | OE1 | 0.147905 |
| | | EP+2 | -0.369598 |
| | | EP-2 | -0.369598 |
| OE2 | -0.812340 | OE2 | 0.147905 |
| | | EP+3 | -0.369598 |
| | | EP-3 | -0.369598 |
| C | 0.692408 | C | 0.539877 |
| O | -0.616991 | O | 0.017428 |
| | | EP+1 | -0.261537 |
| | | EP-1 | -0.261537 |
| | **Aspartate** | | |
| N | -0.756298 | N | -0.763484 |

| | | | |
|---|---|---|---|
| H | 0.328416 | H | 0.354540 |
| CA | 0.113297 | CA | 0.115410 |
| HA | 0.160166 | HA | 0.169097 |
| CB | -0.167596 | CB | 0.010446 |
| HB2 | 0.024299 | HB2 | 0.024862 |
| HB3 | 0.024299 | HB3 | 0.024862 |
| CG | 0.946202 | CG | 0.254448 |
| OD1 | -0.834253 | OD1 | 0.321168 |
| | | EP+2 | -0.442113 |
| | | EP-2 | -0.442113 |
| OD2 | -0.834253 | OD2 | 0.321168 |
| | | EP+3 | -0.442113 |
| | | EP-3 | -0.442113 |
| C | 0.560599 | C | 0.378814 |
| O | -0.564879 | O | 0.051607 |
| | | EP+1 | -0.247243 |
| | | EP-1 | -0.247243 |
| **Glutamine** | | | |
| N | -0.682793 | N | -0.755351 |
| H | 0.350114 | H | 0.387719 |
| CA | -0.022417 | CA | 0.030970 |
| HA | 0.121897 | HA | 0.121812 |
| CB | -0.029233 | CB | -0.034917 |
| HB2 | 0.063041 | HB2 | 0.060355 |
| HB3 | 0.063041 | HB3 | 0.060355 |
| CG | -0.150655 | CG | -0.089306 |
| HG2 | 0.044707 | HG2 | 0.046883 |
| HG3 | 0.044707 | HG3 | 0.046883 |
| CD | 0.824478 | CD | 0.528456 |
| OE1 | -0.670600 | OE1 | -0.002312 |
| | | EP+2 | -0.260920 |
| | | EP-2 | -0.260920 |
| NE2 | -0.998395 | NE2 | -0.842063 |
| HE21 | 0.440747 | HE21 | 0.407452 |
| HE22 | 0.440747 | HE22 | 0.407452 |

| | | | |
|---|---|---|---|
| C | 0.677120 | C | 0.566932 |
| O | -0.516505 | O | -0.025418 |
| | | EP+1 | -0.197031 |
| | | EP-1 | -0.197031 |
| **Asparagine** | | | |
| N | -0.818266 | N | -0.847976 |
| H | 0.431191 | H | 0.451542 |
| CA | 0.015689 | CA | 0.006041 |
| HA | 0.149543 | HA | 0.164574 |
| CB | -0.283303 | CB | -0.128210 |
| HB2 | 0.100889 | HB2 | 0.079323 |
| HB3 | 0.100889 | HB3 | 0.079323 |
| CG | 1.020677 | CG | 0.668810 |
| OD1 | -0.678941 | OD1 | 0.007457 |
| | | EP+2 | -0.265544 |
| | | EP-2 | -0.265544 |
| ND2 | -1.198126 | ND2 | -0.986155 |
| HD21 | 0.480515 | HD21 | 0.428524 |
| HD22 | 0.480515 | HD22 | 0.428524 |
| C | 0.742199 | C | 0.627923 |
| O | -0.543472 | O | 0.001741 |
| | | EP+1 | -0.225177 |
| | | EP-1 | -0.225177 |
| **Histidine (HIE state)** | | | |
| N | -0.707833 | N | -0.673708 |
| H | 0.350429 | H | 0.341525 |
| CA | -0.118901 | CA | -0.143296 |
| HA | 0.119894 | HA | 0.147683 |
| CB | 0.209102 | CB | -0.036344 |
| HB2 | 0.057808 | HB2 | 0.123191 |
| HB3 | 0.057808 | HB3 | 0.123191 |
| CG | 0.043331 | CG | 0.000511 |
| ND1 | -0.602935 | ND1 | 0.750273 |
| | | EP21 | -0.985622 |
| CE1 | 0.208554 | CE1 | 0. 032377 |

| | | | |
|---|---|---|---|
| HE1 | 0.177379 | HE1 | 0. 195482 |
| NE2 | -0.382473 | NE2 | -0.462307 |
| HE2 | 0.323602 | HE2 | 0.383779 |
| CD2 | 0.063033 | CD2 | -0.102355 |
| HD2 | 0.041454 | HD2 | 0.114394 |
| C | 0.760819 | C | 0.730600 |
| O | -0.601071 | O | -0.014726 |
| | | EP+1 | -0.262324 |
| | | EP-1 | -0.262324 |
| **Histidine (HID state)** | | | |
| N | -0.767585 | N | -0.804036 |
| H | 0.394737 | H | 0.422881 |
| CA | -0.058966 | CA | -0.016400 |
| HA | 0.136588 | HA | 0.127834 |
| CB | -0.108693 | CB | -0.047121 |
| HB2 | 0.094819 | HB2 | 0.092793 |
| HB3 | 0.094819 | HB3 | 0.092793 |
| CG | 0.043331 | CG | -0.026425 |
| ND1 | -0.305373 | ND1 | -0.469971 |
| HD1 | 0.338677 | HD1 | 0.419451 |
| CE1 | 0.208554 | CE1 | 0.044763 |
| HE1 | 0.131981 | HE1 | 0.171860 |
| NE2 | -0.602935 | NE2 | 0.797775 |
| | | EP21 | -1.033835 |
| CD2 | 0.063033 | CD2 | -0.114868 |
| HD2 | 0.134017 | HD2 | 0.167909 |
| C | 0.825673 | C | 0.701662 |
| O | -0.622677 | O | -0.001193 |
| | | EP+1 | -0.262936 |
| | | EP-1 | -0.262936 |
| **Tyrosine (*sp*$^2$)** | | | |
| N | -0.761937 | N | -0.805355 |
| H | 0.389733 | H | 0.420181 |
| CA | -0.034787 | CA | 0.019751 |
| HA | 0.134237 | HA | 0.115700 |

| | | | |
|---|---|---|---|
| CB | -0.04492 | CB | -0.032844 |
| HB2 | 0.070234 | HB2 | 0.062867 |
| HB3 | 0.070234 | HB3 | 0.062867 |
| CG | 0.02709 | CG | 0.0334767 |
| CD1 | -0.172006 | CD1 | -0.165339 |
| HD1 | 0.150039 | HD1 | 0.141810 |
| CE1 | -0.202585 | CE1 | -0.148310 |
| HE1 | 0.157342 | HE1 | 0.149371 |
| CZ | 0.246838 | CZ | 0. 031089 |
| OH | -0.54875 | OH | -0.016274 |
| | | EP21 | -0.326425 |
| HH | 0.412294 | HH | 0.326884 |
| CE2 | -0.202585 | CE2 | -0.148310 |
| HE2 | 0.157342 | HE2 | 0.149371 |
| CD2 | -0.172006 | CD2 | -0.165339 |
| HD2 | 0.150039 | HD2 | 0.141810 |
| C | 0.788008 | C | 0.676862 |
| O | -0.613854 | O | 0.000084 |
| | | EP+1 | -0.262610 |
| | | EP-1 | -0.262610 |
| **Tyrosine (*sp*$^3$)** | | | |
| | | N | -0.802499 |
| | | H | 0.418274 |
| | | CA | 0.019196 |
| | | HA | 0.115350 |
| | | CB | -0.024854 |
| | | HB2 | 0.061479 |
| | | HB3 | 0.061479 |
| | | CG | 0.033283 |
| | | CD1 | -0.175104 |
| | | HD1 | 0.148891 |
| | | CE1 | -0.178668 |
| | | HE1 | 0.153794 |
| | | CZ | 0.132394 |
| | | OH | -0.018206 |

| | | | |
|---|---|---|---|
| | | EP+2 | -0.194325 |
| | | EP-2 | -0.194325 |
| | | HH | 0.344061 |
| | | CE2 | -0.178668 |
| | | HE2 | 0.153794 |
| | | CD2 | -0.175104 |
| | | HD2 | 0.148891 |
| | | C | 0.675891 |
| | | O | 0.000788 |
| | | EP+1 | -0.262907 |
| | | EP-1 | -0.262907 |
| **Serine** | | | |
| N | -0.708549 | N | -0.752784 |
| H | 0.381975 | H | 0.409710 |
| CA | 0.020266 | CA | 0.084222 |
| HA | 0.192246 | HA | 0.171601 |
| CB | 0.072273 | CB | 0.032613 |
| HB2 | 0.075861 | HB2 | 0.060899 |
| HB3 | 0.075861 | HB3 | 0.060899 |
| OG | -0.649230 | OG | -0.024236 |
| HG | 0.440238 | HG | 0.361709 |
| | | EP+2 | -0.244436 |
| | | EP-2 | -0.244436 |
| C | 0.691544 | C | 0.598567 |
| O | -0.592484 | O | 0.001621 |
| | | EP+1 | -0.257975 |
| | | EP-1 | -0.257975 |
| **Threonine** | | | |
| N | -0.760940 | N | -0.837103 |
| H | 0.397030 | H | 0.421759 |
| CA | 0.123464 | CA | 0.304277 |
| HA | 0.175883 | HA | 0.118985 |
| CB | 0.136428 | CB | 0.089172 |
| HB | 0.054611 | HB | 0.033819 |
| CG2 | -0.14856 | CG2 | -0.111582 |

| | | | |
|---|---|---|---|
| HG21 | 0.058564 | HG21 | 0.043062 |
| HG22 | 0.058564 | HG22 | 0.043062 |
| HG23 | 0.058564 | HG23 | 0.043062 |
| OG1 | -0.666196 | OG1 | -0.041629 |
| HG1 | 0.428784 | HG1 | 0.349871 |
| | | EP+2 | -0.247979 |
| | | EP-2 | -0.247979 |
| C | 0.675438 | C | 0.548043 |
| O | -0.591634 | O | -0.008613 |
| | | EP+1 | -0.250113 |
| | | EP-1 | -0.250113 |
| **Lysine** | | | |
| N | -0.470293 | N | -0.488756 |
| H | 0.250043 | H | 0.258437 |
| CA | -0.041329 | CA | 0.008918 |
| HA | 0.107966 | HA | 0.099785 |
| CB | -0.017259 | CB | -0.010584 |
| HB2 | 0.011460 | HB2 | 0.009803 |
| HB3 | 0.011460 | HB3 | 0.009803 |
| CG | -0.015464 | CG | -0.019098 |
| HG2 | 0.005667 | HG2 | 0.013106 |
| HG3 | 0.005667 | HG3 | 0.013106 |
| CD | 0.009944 | CD | -0.001239 |
| HD2 | 0.036693 | HD2 | 0.037163 |
| HD3 | 0.036693 | HD3 | 0.037163 |
| CE | 0.067301 | CE | 0.060503 |
| HE2 | 0.108924 | HE2 | 0.106623 |
| HE3 | 0.108924 | HE3 | 0.106623 |
| NZ | -0.660955 | NZ | -0.597710 |
| HZ1 | 0.405830 | HZ1 | 0.389236 |
| HZ2 | 0.405830 | HZ2 | 0.389236 |
| HZ3 | 0.405830 | HZ3 | 0.389236 |
| C | 0.877101 | C | 0.759068 |
| O | -0.650029 | O | -0.016678 |
| | | EP+1 | -0.276873 |

| | | | |
|---|---|---|---|
| | | EP-1 | -0.276873 |
| **Arginine** | | | |
| N | -0.451629 | N | -0.489554 |
| H | 0.260148 | H | 0.264567 |
| CA | -0.060903 | CA | -0.017389 |
| HA | 0.104496 | HA | 0.107680 |
| CB | -0.021906 | CB | -0.009199 |
| HB2 | 0.017296 | HB2 | 0.015842 |
| HB3 | 0.017296 | HB3 | 0.015842 |
| CG | 0.031084 | CG | 0.020851 |
| HG2 | 0.005058 | HG2 | 0.014496 |
| HG3 | 0.005058 | HG3 | 0.014496 |
| CD | 0.029314 | CD | -0.004762 |
| HD2 | 0.095248 | HD2 | 0.105133 |
| HD3 | 0.095248 | HD3 | 0.105133 |
| NE | -0.569153 | NE | -0.550419 |
| HE | 0.389115 | HE | 0.387120 |
| CZ | 0.793842 | CZ | 0.775401 |
| NH1 | -0.875346 | NH1 | -0.866861 |
| HH11 | 0.447645 | HH11 | 0.445699 |
| HH12 | 0.447645 | HH12 | 0.445699 |
| NH2 | -0.875346 | NH2 | -0.866861 |
| HH21 | 0.447645 | HH21 | 0.445699 |
| HH22 | 0.447645 | HH22 | 0.445699 |
| C | 0.860863 | C | 0.735457 |
| O | -0.640365 | O | -0.029726 |
| | | EP+1 | -0.255022 |
| | | EP-1 | -0.255022 |
| **Tryptophan** | | | |
| N | -0.775714 | N | -0.791969 |
| H | 0.396418 | H | 0.414489 |
| CA | -0.016596 | CA | 0.027656 |
| HA | 0.144761 | HA | 0.132769 |
| CB | -0.101428 | CB | -0.066863 |
| HB2 | 0.101584 | HB2 | 0.093188 |

| | | | |
|---|---|---|---|
| HB3 | 0.101584 | HB3 | 0.093188 |
| CG | -0.076610 | CG | -0.098367 |
| CD1 | -0.081668 | CD1 | -0.057900 |
| HD1 | 0.125415 | HD1 | 0.104909 |
| NE1 | -0.459363 | NE1 | -0.457924 |
| HE1 | 0.386072 | HE1 | 0.385290 |
| CE2 | 0.115048 | CE2 | 0.110693 |
| CZ2 | -0.163021 | CZ2 | -0.159764 |
| HZ2 | 0.130626 | HZ2 | 0.129805 |
| CH2 | -0.157693 | CH2 | -0.158941 |
| HH2 | 0.149862 | HH2 | 0.149856 |
| CZ3 | -0.203775 | CZ3 | -0.203544 |
| HZ3 | 0.151567 | HZ3 | 0.151606 |
| CE3 | -0.129678 | CE3 | -0.124226 |
| HE3 | 0.142544 | HE3 | 0.139374 |
| CD2 | 0.048724 | CD2 | 0.046696 |
| C | 0.791474 | C | 0.669067 |
| O | -0.620131 | O | -0.006476 |
| | | EP+1 | -0.261305 |
| | | EP-1 | -0.261305 |

**Supplementary Table S2.** The number of possible hydrogen bonds formed between the polar atoms of the hydrophilic amino acids and hydration water molecules.

| Amino acid | Atom | Hybridization | Number of lone pairs/bonds with H | Number of possible hydrogen bonds |
|---|---|---|---|---|
| Peptide bond | O | $sp^2$ | 2/0 | 2 |
| | N | $sp^2$ | 0/1 | 1 |
| Glu | OE1 | $sp^2$ | 2/0 | 2 |
| | OE2 | $sp^2$ | 2/0 | 2 |
| Asn | OD1 | $sp^2$ | 2/0 | 2 |
| | OD2 | $sp^2$ | 2/0 | 2 |
| Gln | OE1 | $sp^2$ | 2/0 | 2 |
| | NE2 | $sp^2$ | 0/2 | 2 |
| Asn | OD1 | $sp^2$ | 2/0 | 2 |
| | ND2 | $sp^2$ | 0/2 | 2 |
| His (HIE) | ND1 | $sp^2$ | 1/0 | 1 |
| | NE2 | $sp^2$ | 0/1 | 1 |
| His (HID) | ND1 | $sp^2$ | 0/1 | 1 |
| | NE2 | $sp^2$ | 1/0 | 1 |
| Tyr | OH | $sp^2$ | 1/1 | 2 |
| | OH | $sp^3$ | 2/1 | 3 |
| Ser | OG | $sp^3$ | 2/1 | 3 |
| Thr | OG1 | $sp^3$ | 2/1 | 3 |
| Lys | NZ | $sp^3$ | 0/3 | 3 |
| Arg | NE | $sp^2$ | 0/1 | 1 |
| | NH1 | $sp^2$ | 0/2 | 2 |
| | NH2 | $sp^2$ | 0/2 | 2 |
| Trp | NE1 | $sp^2$ | 0/1 | 1 |

**Supplementary Table S3.** The relative root-mean-square (RRMS) errors of ESP that are calculated from the charges determined by the RESP procedure using the restraint strength of 0.001 au. The values in brackets are the RRMS errors when using the restraint strength of 0.0005 au.

| Peptide | RRMS[a] (without LP electrons) | RRMS[a] (with LP electrons) |
|---|---|---|
| GEKG | 0.0224 (0.0220) | 0.0236 (0.0225) |
| GERG | 0.0224 (0.0217) | 0.0235 (0.0223) |
| GDKG | 0.0338 (0.0329) | 0.0306 (0.0298) |
| GQG | 0.0272 (0.0266) | 0.0285 (0.0274) |
| GNG | 0.0269 (0.0262) | 0.0284 (0.0273) |
| GHG (HIE state) | 0.0362 (0.0353) | 0.0327 (0.0316) |
| GHG (HID state) | 0.0296 (0.0286) | 0.0336 (0.0331) |
| GYG (*sp*$^2$) | 0.0280 (0.268) | 0.0290 (0.0278) |
| GYG (*sp*$^3$) | | 0.0307 (0.0299) |
| GSG | 0.0257 (0.0252) | 0.0260 (0.0254) |
| GTG | 0.0277 (0.0264) | 0.0288 (0.0274) |
| GWG | 0.0270 (0.0261) | 0.0273 (0.0263) |

[a] $RRMS = \left( \sum_{i=1}^{N_{\mathrm{ESP}}} \left( \hat{V}_i - V_i \right)^2 \Big/ \sum_{i=1}^{N_{\mathrm{ESP}}} V_i^2 \right)^{1/2}$, where $N_{\mathrm{ESP}}$, $V_i$, and $\hat{V}_i$ refer to the number of ESP points, the QM ESP, and ESP calculated from the charges determined by the RESP procedure, respectively.

**Supplementary Table S4.** Atom-centered and off-atom charges of amino acids determined by the standard RESP protocol applied to the blocked di-peptides.

| Without LP electrons | | With LP electrons | |
|---|---|---|---|
| **Charge site** | **Charges** | **Charge site** | **Charges** |
| **Glutamate** | | | |
| N | -0.489110 | N | -0.519193 |
| H | 0.255155 | H | 0.278576 |
| CA | 0.070501 | CA | 0.064514 |
| HA | 0.120070 | HA | 0.132664 |
| CB | 0.015423 | CB | 0.015664 |
| HB2 | 0.004160 | HB2 | 0.010070 |
| HB3 | 0.004160 | HB3 | 0.010070 |
| CG | -0.018505 | CG | 0.033277 |
| HG2 | -0.024488 | HG2 | -0.011041 |
| HG3 | -0.024488 | HG3 | -0.011041 |
| CD | 0.795881 | CD | 0.279730 |
| OE1 | -0.819333 | OE1 | 0.219928 |
| | | EP+2 | -0.411414 |
| | | EP-2 | -0.411414 |
| OE2 | -0.819333 | OE2 | 0.219928 |
| | | EP+3 | -0.411414 |
| | | EP-3 | -0.411414 |
| C | 0.431213 | C | 0.334958 |
| O | -0.501305 | O | 0.025703 |
| | | EP+1 | -0.219075 |
| | | EP-1 | -0.219075 |
| **Lysine** | | | |
| N | -0. 346760 | N | -0.377489 |
| H | 0. 225628 | H | 0.251560 |
| CA | 0.013229 | CA | 0.011366 |
| HA | 0.068248 | HA | 0.074435 |
| CB | -0.025609 | CB | -0.019488 |
| HB2 | 0.022544 | HB2 | 0.021336 |

| | | | |
|---|---|---|---|
| HB3 | 0.022544 | HB3 | 0.021336 |
| CG | 0.012107 | CG | 0.010336 |
| HG2 | 0.013167 | HG2 | 0.013399 |
| HG3 | 0.013167 | HG3 | 0.013399 |
| CD | -0.023662 | CD | -0.022498 |
| HD2 | 0.060025 | HD2 | 0.059861 |
| HD3 | 0.060025 | HD3 | 0.059861 |
| CE | 0.019098 | CE | 0.019392 |
| HE2 | 0.110639 | HE2 | 0.110867 |
| HE3 | 0.110639 | HE3 | 0.110867 |
| NZ | -0.584280 | NZ | -0.592338 |
| HZ1 | 0.393432 | HZ1 | 0.395896 |
| HZ2 | 0.393432 | HZ2 | 0.395896 |
| HZ3 | 0.393432 | HZ3 | 0.395896 |
| C | 0.564585 | C | 0.479401 |
| O | -0.515633 | O | 0.016723 |
| | | EP+1 | -0.225007 |
| | | EP-1 | -0.225007 |

**Supplementary Table S5.** The positions of the peaks in the density distributions.

| Amino acid | Distribution type | Peak positions in MD-LP | Peak positions in MD-noLP |
|---|---|---|---|
| Glu (TIP4P/Ew) | $r$ /Å | 2.65 (0.15) | 2.65 (0.17) |
| | $\theta_x$ /° | -50 (15), 50 (12) | -55 (19), 50 (13) |
| | $\varphi$ /° | 0 (29), 170 (34) | -30 (30) |
| | Total amount of water densities around $COO^-$ group [b] | 4.6 | 4.5 |
| Glu (SPC/E) | $r$ /Å | 2.65 (0.16) | 2.65 (0.17) |
| | $\theta_x$ /° | -45 (17), 55 (11) | -55 (19), 55 (11) |
| | $\varphi$ /° | -5 (25), 165 (33), | 0 (25) |
| | Total amount of water densities around $COO^-$ group [b] | 4.6 | 4.6 |
| Glu (TIP5P) | $r$ /Å | 2.65 (0.18) | 2.75 (0.20) |
| | $\theta_x$ /° | -60 (32), 55 (14) | -60 (53), 50 (14) |
| | $\varphi$ /° | -10 (68), 160 (36) | None |
| | Total amount of water densities around $COO^-$ group [b] | 4.1 | 3.8 |

**References**


1. Dixon, R. W. & Kollman, P. A. Advancing beyond the atom-centered model in additive and nonadditive molecular mechanics. *J. Comput. Chem.* **18**, 1632-1646 (1997).
2. Lu, Z., Zhou, N., Wu, Q. & Zhang, Y. Directional dependence of hydrogen bonds: a density-based energy decomposition analysis and its implications on force field development. *J. Chem. Theory Comput.* **7**, 4038–4049 (2011).
3. Baker, E. N.; Hubbard, R. E. Hydrogen bonding in globular proteins. *Prog. Biophys. Mol. Biol.* **44**, 97-179 (1984).
4. Ippolito, J. A., Alexander, R. S. & Christianson, D. W. Hydrogen bond stereochemistry in protein structure and function. *J. Mol. Biol.* **215**, 457-471 (1990).
5. Matsuoka, D. & Nakasako, M. Probability distributions of hydration water molecules around polar protein atoms obtained by a database analysis. *J. Phys. Chem. B* **113**, 11274-11292 (2009).
6. Cornell, W. D. *et al.* A second generation force field for the simulation of proteins, nucleic acids, and organic molecules. *J. Am. Chem. Soc.* **117**, 5179-5197 (1995).
7. Cieplak, P., Cornell, W. D., Bayly, C. & Kollman P. A. Application of the multimolecule and multiconfomational RESP methodology to biopolymers: charge derivation for DNA, RNA, and proteins. *J. Chem. Comput.* **16***,* 1357-1377 (1995).
8. Jorgensen, W. L., Chandrasekhar, J., Madura, J. D., Impey, R. W. & Klein, M. L. Comparison of simple potential functions for simulating liquid water. *J. Chem. Phys.* **79**, 926-935 (1983).
9. Horn, H. W. *et al.* Development of an improved four-site water model for biomolecular simulations. *J. Chem. Phys.* **120**, 9665-9678 (2004).
10. Mahoney, M. W. & Jorgensen, W. L. A five-site model for liquid water and the reproduction of the density anomaly by rigid, nonpolarizable potential functions. *J. Chem. Phys.* **112**, 8910-8922 (2000).

11. Berendsen, H. J. C., Grigera, J. R. & Straatsma, T. P. The missing term in effective pair potentials. *J. Phys. Chem.* **91**, 6269-6271 (1987).
